# Chronic Electrical Stimulation with a Suprachoroidal Retinal Prosthesis: A Preclinical Safety and Efficacy Study

**DOI:** 10.1371/journal.pone.0097182

**Published:** 2014-05-22

**Authors:** David A. X. Nayagam, Richard A. Williams, Penelope J. Allen, Mohit N. Shivdasani, Chi D. Luu, Cesar M. Salinas-LaRosa, Sue Finch, Lauren N. Ayton, Alexia L. Saunders, Michelle McPhedran, Ceara McGowan, Joel Villalobos, James B. Fallon, Andrew K. Wise, Jonathan Yeoh, Jin Xu, Helen Feng, Rodney Millard, Melanie McWade, Patrick C. Thien, Chris E. Williams, Robert K. Shepherd

**Affiliations:** 1 Bionics Institute, East Melbourne, Victoria, Australia; 2 Department of Pathology, University of Melbourne, Parkville, Victoria, Australia; 3 Department of Anatomical Pathology, St Vincent’s Hospital Melbourne, Fitzroy, Victoria, Australia; 4 Centre for Eye Research Australia, University of Melbourne, East Melbourne, Victoria, Australia; 5 Department of Ophthalmology, The University of Melbourne, East Melbourne, Victoria, Australia; 6 Statistical Consulting Centre, The University of Melbourne, Parkville, Victoria, Australia; 7 The HEARing Cooperative Research Centre, The University of Melbourne, East Melbourne, Victoria, Australia; 8 Department of Otolaryngology, The University of Melbourne, East Melbourne, Victoria, Australia; 9 Biomedical Engineering Department, Vanderbilt University, Nashville, Tennessee, United States of America; 10 Medical Bionics Department, University of Melbourne, East Melbourne, Victoria, Australia; Monash University, Australia

## Abstract

**Purpose:**

To assess the safety and efficacy of chronic electrical stimulation of the retina with a suprachoroidal visual prosthesis.

**Methods:**

Seven normally-sighted feline subjects were implanted for 96–143 days with a suprachoroidal electrode array and six were chronically stimulated for 70–105 days at levels that activated the visual cortex. Charge balanced, biphasic, current pulses were delivered to platinum electrodes in a monopolar stimulation mode. Retinal integrity/function and the mechanical stability of the implant were assessed monthly using electroretinography (ERG), optical coherence tomography (OCT) and fundus photography. Electrode impedances were measured weekly and electrically-evoked visual cortex potentials (eEVCPs) were measured monthly to verify that chronic stimuli were suprathreshold. At the end of the chronic stimulation period, thresholds were confirmed with multi-unit recordings from the visual cortex. Randomized, blinded histological assessments were performed by two pathologists to compare the stimulated and non-stimulated retina and adjacent tissue.

**Results:**

All subjects tolerated the surgical and stimulation procedure with no evidence of discomfort or unexpected adverse outcomes. After an initial post-operative settling period, electrode arrays were mechanically stable. Mean electrode impedances were stable between 11–15 kΩ during the implantation period. Visually-evoked ERGs & OCT were normal, and mean eEVCP thresholds did not substantially differ over time. In 81 of 84 electrode-adjacent tissue samples examined, there were no discernible histopathological differences between stimulated and unstimulated tissue. In the remaining three tissue samples there were minor focal fibroblastic and acute inflammatory responses.

**Conclusions:**

Chronic suprathreshold electrical stimulation of the retina using a suprachoroidal electrode array evoked a minimal tissue response and no adverse clinical or histological findings. Moreover, thresholds and electrode impedance remained stable for stimulation durations of up to 15 weeks. This study has demonstrated the safety and efficacy of suprachoroidal stimulation with charge balanced stimulus currents.

## Introduction

The retina is a delicate multi-layered tissue in the back of the eye responsible for the initiation and first-order processing of vision [1]. Certain pathologies, such as retinitis pigmentosa (RP; the major inherited form of blindness affecting 1.5 million people worldwide), lead to progressive degeneration of the retinal photoreceptors, the cells responsible for transducing light into neural impulses [2]. This ultimately results in profound vision loss or blindness. Importantly, significant numbers of neurons in the inner retina are spared following the loss of photoreceptors [3], [4]. Retinal prostheses can restore visual perception to blind RP patients by using targeted electrical stimulation to directly stimulate these residual neurons, particularly the retinal ganglion cells [5]–[7]. An array of implanted electrodes, mapped across the visual field, can be used to create phosphenes (visual percepts not evoked by light). In this manner, retinal prostheses can assist in navigation and object recognition tasks, taking advantage of the remaining neural processing within the central visual pathway [8], [9].

A primary concern for all medical devices is one of safety and, as such, preclinical studies are essential to ensure that patient risk is minimised. In the case of retinal prostheses, chronic electrical stimulation studies are an important part of a preclinical workflow that culminates in a clinical trial. However, due to the technical complexity of assessing the safety of electrically stimulating the retina in an *in vivo* model, there have been few preclinical studies performed [10]–[17] (Table 1). To our knowledge, none have used chronic and continuous electrical stimulation delivery combined with electrophysiological verification of thresholds, clinical monitoring and histological endpoints. Therefore, clinical retinal prostheses have largely relied on electrical stimulus safety limits derived from other devices [18], [19], and/or calculated from *in vitro* electrochemistry [20]–[23]. A judicious preclinical workflow should ideally include a chronic electrical stimulation safety study performed using the clinical electrode array design, implanted in the desired anatomical location [24].

**Table 1:** Summary of parameters used in previous preclinical retinal stimulation studies.

**Study**	*Güven et al (2005)*		*Gekeler et al (2007)*		*Terasawa et al (2013)*	
**Species**	Normal & Blind Dog		Pig		Rabbit	
**Location**	Epiretinal		Subretinal		Intra-Scleral	
**Duty Cycle | Stimulation** **Duration**	∼9 hrsper day	∼3 months	1 hr per day	1 month	8 hrsper day	1 month
**Electrode** **Array | Maximum Charge** **Density Per** **Phase**	4×4 Pt	≤100 µC cm^−2^	1,550 MPDA + 4×4 Au	<2 V*	1x Pt Bullet	∼180 µC cm^−2^
**Assessment Tools**	Fundus, ERG, eEVCP, Histopathology		Fundus, OCT, FA,Histopathology		Fundus, OCT, eEVCP,Histopathology	

*N.B. The Gekeler et al (2007) study used a micro-photodiode array (MPDA) to provide electrical energy from incident photons, so it is not possible to provide an estimate of charge density.

Abbreviations: ERG, electroretinography; eEVCP, electrically-evoked visual cortex potential recordings; OCT, optical coherence tomography; FA, fluorescein angiography.

The suprachoroidal space is located between the sclera and the choroid. It is a surgically accessible cleavage plane capable of housing a relatively large electrode array without penetrating the neural retina or exposing the vitreous chamber. This location provides a device implanted in this space with mechanical stability. The suprachoroidal approach also reduces the risk of surgical trauma and eliminates a direct pathway for retinal infection [25].

Chronic implantation safety (biocompatibility) of a passive (unstimulated) silicone and platinum array located in the suprachoroidal space has previously been demonstrated [26]. The present study aimed to evaluate any additional safety (i.e. induced damage/pathology) or efficacy (i.e. functional) concerns that may be associated with the introduction of chronic electrical stimulation in a preclinical feline model. The present study is the first to investigate the biological response to chronic suprathreshold stimulation of the retina from the suprachoroidal space.

## Methods

We assessed the safety of chronic continuous electrical stimulation delivered to the retina via an electrode array located in the suprachoroidal space. We used standard clinical tools (fundus inspection, optical coherence tomography [OCT] and electroretinography [ERG]) to evaluate the structure and function of the eye during the implantation and chronic stimulation period. Electrode impedances were regularly measured to assess the stability of the electrode-tissue interface. At the completion of the study, the retinal tissue was evaluated histopathologically and immunohistochemically for evidence of stimulation induced injury. Furthermore, by integrating a cortical recording system and performing regular electrophysiology, the efficacy and functional stability of retinal stimulation from a suprachoroidal array was also examined over time. Table 2 summarises the study design employed in the present study, described in more detail below.

**Table 2:** Summary of study design.

**N = **	7	
**Species**	Cat	
**Location**	Suprachoroidal	
**Controls**	Non-implanted Contralateral Eye and Unstimulated Electrodes	
**Duty Cycle | Stimulation Duration**	24 hrs per day	∼3 months
**Electrode Array**	12× Pt 600 µm diameter discs (actives) + 2× Pt 2 mm diameter discs (returns); Conformable Silicone Carrier	
**Maximum Charge Density Per Phase**	≤77 µC.cm^−2^	
**Stimulation**	Charge-Balanced Biphasic Current Pulses	
**Assessment Tools**	Fundus, OCT, ERG, X-Ray, eEVCP, Cortical Multi-Unit Recordings, Histopathology	

*Abbreviations: OCT, optical coherence tomography; ERG, electroretinography; eEVCP, electrically-evoked visual cortex potential recordings.*

### Ethics Statement

All procedures were approved by The Royal Victorian Eye and Ear Hospital’s Animal Research & Ethics Committee (RVEEH AEC; #10/206AB). The subjects were treated according to the National Health and Medical Research Council’s “Australian Code of Practice for the Care and Use of Animals for Scientific Purposes” (2013) and the “Prevention of Cruelty to Animals Act” (1986; and amendments). All surgical, clinical assessment and electrophysiological procedures were carried out under anaesthesia and all efforts were made to minimise suffering.

### Implant System

The retinal stimulation system used in the present study was based on previous chronic cochlear implant stimulation studies [27], [28]. The system included a suprachoroidal electrode array, a transcutaneous lead with integrated extradural recording electrodes, a wearable external stimulator with processor, and a “backpack” harness that housed the external components as well as protected the location where the lead exited the body.

**Figure 1:**
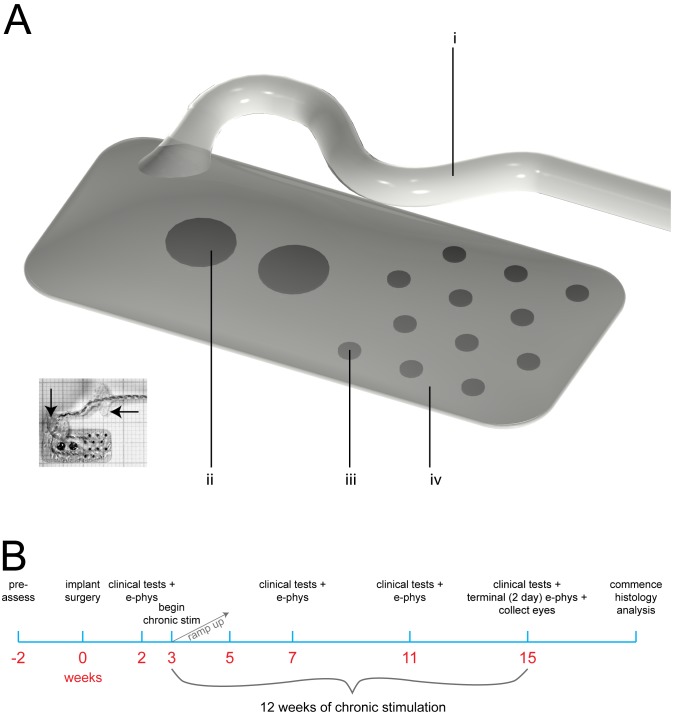
Study design. (**A**) Schematic Drawing of the suprachoroidal electrode array used in the present study: (i) fourteen individually insulated platinum (Pt) – iridium (Ir) wires (25 µm diameter) are coiled into a helix and encased within a medical grade silicone tube; (ii) two 2 mm diameter Pt disc electrodes; (iii) twelve 600 µm diameter Pt disc electrodes welded to Pt/Ir wires; iv, spherically conformable medical grade silicone base; viii. **(Inset)** shows an ∼1∶1 scale photograph of an array on a 1 mm grid. Visible in the photograph is the 25 µm paralyne-insulated Pt-Ir wires which were resistance welded to each electrode. Also visible are two silicone-coated Polyethylene terephthalate (Dacron) patches (arrows) used for fixation at the sclera and orbital bone. (**B**) Study timeline for a single subject. Abbreviations: e-phys, electrophysiology; stim, stimulation. Individual alterations to timeline are described in Table 3.

The electrode array was manufactured using medical grade silicone and platinum (Pt). It was similar to previously published designs [29] and was a precursor to a clinical prototype (www.clincialtrials.gov #NCT01603576; Figure 1A) [25]. The array used in the present preclinical study was 17 mm×8 mm with a maximum thickness of 1 mm. It was rounded at the corners, conformable and contoured for minimal insertion and implantation trauma [30]. Twelve 600 µm diameter Pt disc electrodes were positioned in four rows of three electrodes each. The electrodes were arranged in an offset (hexagonal) layout with a 2 mm pitch. Additionally, two 2 mm diameter Pt discs were used as large surface area return electrodes for stimulating in monopolar configuration. Individually insulated platinum-iridium (Pt-Ir) wires were welded to the fourteen electrodes in the array and these wires were coiled together into a helical silicone lead of 1.2 mm diameter. Two Dacron mesh patches, embedded in medical grade silicone were used as suture sites designed to stabilise the electrode array and intraorbital lead. These patches were located at the scleral wound and at the orbital notch (Fig. 1A).

The fourteen Pt wires in the lead were individually connected to more robust insulated stainless steel wires, via an implanted connector that was secured within the feline bulla cavity. This stainless steel cable was secured with titanium clips to the subject’s skull. The lead system also incorporated two stainless steel wire recording electrodes as well as third stainless steel reference electrode. These recording electrodes were implanted extradurally over the contralateral visual cortex, with the reference electrode at the nape of the neck, in order to record evoked cortical activity. The entire implant was cleaned and sonicated in ethanol and liquid pyroneg detergent solutions (Diversey A/Asia Pty Ltd, NSW, Australia) before being rinsed in sterile water and sterilised in an autoclave for 44 min at 3100 mbar/135°C.

In a similar fashion to previous chronic stimulation studies [31], a Nucleus (CI24RE) cochlear implant (Cochlear) was modified for use as a wearable multichannel stimulator. The device used to power and control the cochlear implant stimulator was a Freedom behind the ear processor (Cochlear), which was reprogrammed to provide customisable stimulation (see below for chronic stimulation parameters used in the present study).

Elasticised cotton backpack harnesses were customised and hand-fabricated for each subject for comfort. The external components, including lead, connector, stimulator and processor were securely housed in a dorsal pocket with a Velcro closure. A larger flap (also with Velcro closure) provided access to the surgical defect created in the skin, between the scapulae, to permit the lead to exit the body. The entire wearable system (including the backpack harness) weighed approximately 90 g.

### Cohort and Timeline

Seven normal healthy adult *Felis catus* were used for the present study; they were assigned identification numbers (502–508). Each subject’s experiment schedule followed a timeline similar to that shown in Figure 1B. Individual variations in each subject’s stimulation regime are detailed in Table 3. Two weeks prior to implantation, each subject underwent a health examination, including external ocular health, and baseline clinical eye assessments were performed (see below for details). Clinical assessments were repeated two weeks post-implantation and then monthly after the commencement of chronic stimulation. In addition, electrophysiological recordings were performed during the same anaesthetised sessions as the clinical assessments from two weeks post-implantation onwards. At three weeks post-surgery, when the subject was fully recovered, the subject’s backpack stimulators were activated at a low stimulus intensity which was slowly increased to the maximum levels deliverable (refer to Table 3) over the course of the next two weeks. After three months of continuous stimulation (duration chosen to facilitate comparisons with previous chronic implantation studies [10], [26]) the subjects underwent a final clinical assessment before commencing a 48–72 hour acute electrophysiology session. Following this, subjects were overdosed with barbiturate anaesthetic and transcardially perfused. Both eyes were prepared for histopathological evaluation [32].

**Table 3:** Summary of chronic stimulation cohort.

Subject ID#	Implant Duration (days)	Stimulation Duration (days)	Max ChargeDensity PerPhase (µC.cm^−2^)	Electrode Utilisation		
				Max	Partial	None
**502**	143	98	45	7	5	0
**503**	96	70	77	11	1	0
**504**	107	74	45	7	4	1
**505**	107	18*	22	1	1	10
**506**	128	105	53	11	0	1
**507**	114	79	53	10	1	1
**508**	135	102	37	9	2	1

*Stimulation mode was monopolar. Stimulation was delivered using biphasic charge balanced rectangular current pulses (phase width: 400 µs; interphase gap: 20 µs). The maximum current denotes the stimulation level at the culmination of the ramp-up period (refer Figure 1). Charge density per phase was calculated by using the stimulation phase width of 400 µs per phase and the geometric electrode surface area of 0.2827 mm^2^. Electrode utilisation, expressed as “X|Y|Z” denotes the number of electrodes that were stimulated maximally during the stimulation period (X); the number of electrodes that were partially stimulated, either at lower levels or for an incomplete subset of the stimulation duration (Y); and the number of unstimulated controls on the array (Z). The lead system was damaged during subject 505’s implantation; therefore it was only stimulated for 18 days (asterisk) before being disconnected from the stimulator.*

### Anaesthesia and Monitoring

The anaesthetic and monitoring procedures used in the present study are similar to previously published reports [26]. Briefly, for clinical assessments and electrophysiology sessions a deep anaesthetic state was induced with subcutaneous xylazine (2 mg/kg s.c.; Ilium Xylazil-20, Troy Laboratories, NSW, Australia) and ketamine (20 mg/kg s.c.; Ilium Ketamil, Troy Laboratories, NSW, Australia). For implant surgery, xylazine and ketamine (dosage as above) was used to induce a deep anaesthetic state and this was maintained with gaseous Isoflurane (Abbott Australasia, Pty Ltd Australia; 1–3%) delivered via an endotracheal tube. For the terminal electrophysiology session, subjects were induced into a deep anaesthetic state with xylazine and ketamine (dosage as above) and were fitted bilaterally with intravenous catheters in the cephalic vein, connected to automated syringe drivers (Terumo, Terfusions syringe pump TE-331, Japan) which maintained pentobarbitone anaesthesia (0.2–2.0 mL/h; Ilium, Troy Laboratories Pty Ltd, Australia) and hydration (Hartmann’s Solution; 2.5 ml/kg/h). The subject’s breathing and blood pressure were monitored regularly during all procedures (Cardell veterinary monitor 9405, Casmed Medical Systems, USA) and body temperature was maintained at 37°C. Clinical assessments, electrophysiology sessions (excluding the terminal session), and implant surgeries all lasted approximately 3–5 hours. During these procedures, subject’s pupils were dilated with 1% tropicamide (Chauvin Pharmaceuticals, Surrey, England) and 2.5% phenylephrine hydrochloride (Chauvin Pharmaceuticals, Surrey, England). At the completion of the procedures (excluding the terminal electrophysiology session) subjects were rehydrated with Hartmann’s solution (2.5 ml/kg/h; s.c.) and allowed to wake naturally.

### Implantation Surgery

The procedures for implanting the suprachoroidal electrode array have been described previously [25], [26]. However, the lead routing required for the current study has not been described before. All suprachoroidal implant surgeries were performed by retinal surgeons under aseptic conditions. Subjects were deeply anaesthetised, intubated and monitored as above. The planned incision sites were shaved and skin disinfected with povidone-iodine (Betadine; antiseptic solution, Mundipharma, Netherlands). A full-thickness midline incision was made over the head and then the skin and the underlying muscle was retracted to expose the cranium. The silicone coated stainless steel lead was tunnelled with a trocar superocaudally from a lateral canthotomy wound in the left eye to the skull, where it was secured with titanium bone screws. The array was then implanted in the suprachoroidal space as previously described [25], [26]. The connector end was tunnelled subcutaneously from the skull, posteriorly, and exited at a wound created between the subject’s scapulae. Surgical wounds were closed in layers with a combination of sutures (Ethicon, Mexico) and staples (Smith and Nephew Medical Ltd, Germany). Closed surgical wounds were liberally sprayed with OpSite (Smith and Nephew Medical Ltd, England). The position of the implant array in the suprachoroidal space was visible as a shadow when viewed in a fundus image (Figure S1), produced by the change in light reflection angle from the elevated choroid and tapetum (a reflective tissue layer in the feline eye located between the choroid and the retina), at the edge of the array. The retina and implant position was photographed using a fundus lens (Volk® Quadrasheric fundus lens, Ohio USA) and surgical microscope (Zeiss OPMI 6-CFR XY, Carl Zeiss AG) fitted with a beam splitter and camera mount (Carl Zeiss AG).

### Postoperative Care

Subjects were monitored daily throughout their implantation period, by staff experienced in animal-husbandry, and were regularly checked by a veterinarian. Analgesic (buprenophrine 0.01 mg/kg, SC; Temgesic; Reckitt Benckiser, Sydney, Australia Temgesic) was administered intra-operatively at the completion of the procedure and again the following day. For the first week post-operatively the subjects was given amoxicillin-clavulanate suspension once daily (10 mg/kg, SC; Clavulox; Pfizer Italia, Rome, Italy). For several days after surgeries, local and systemic antibiotics (respectively: Chlorsig; Sigma Pharmaceuticals, VIC, Australia; Noroclav; Norbrook, Newry, Northern Ireland), corticosteroids (Predneferin Forte; Sigma Pharmaceuticals, VIC, Australia) and anti-cholinergic drugs (1% atropine sulphate; Chauvin Pharmaceuticals, Surrey, England) were administered regularly as deemed necessary by the surgeons and/or veterinarian. The lead exit wound was cleaned and disinfected daily until fibrous encapsulation was achieved (approximately 2–3 weeks), after which it, and the other surgical wounds, were inspected daily and cleaned every few days.

### Electrode Impedance Measurements

Electrode impedance was measured at the end of the cathodic (first) phase of a biphasic current pulse and defined as the peak voltage divided by the current [33]. Individual electrode impedances were measured using commercial clinical software (Custom Sound EP; Cochlear) running on a laptop computer. Impedance measurements were calculated and averaged in response to trains of 25 µs phase-width rectangular biphasic current pulses of 75 µA amplitude. Measurements were made intraoperatively to ensure that the implant was functional before closing the surgical wounds. After surgical recovery, impedance measurements were made regularly (approximately weekly) for the duration of the experiment. Impedances during the implantation period were compared with impedance measurements made in 0.9% saline: prior to implantation; after termination and removal; and after a final cleaning (i.e. sonication in ethanol and liquid pyroneg detergent solutions). Although this measure does not include a phase angle – and thus could correctly be termed ‘mean of peak cathodic-phase instantaneous resistances’, the term ‘impedance’ has become commonly accepted in many clinical neuroprosthesis studies and we have adopted it here for simplicity. Non-stimulated control electrodes were selected ad-hoc, following surgery and prior to the commencement of stimulation based on impedance results. This selection of open circuit channels was predicated on the expected 10–20% random attrition margin due to failures in the lead system and connector [34]).

### Clinical Assessments

The effect of chronic implantation on retinal structure was investigated using colour fundus photography (TRC-50Dx, Topcon Medical Systems, NJ, USA) and Fourier domain OCT (Spectralis, Heidelberg Engineering GmbH, Heidelberg, Germany). Retinal function was assessed using full-field ERG (Espion E2, Diagnosys LLC, Lowell, MA, USA). Clinical assessment tests have been used previously in preclinical studies evaluating the safety of retinal prostheses [10], [15], [16], [26], and are also common clinical ophthalmological tests.

#### Fundus photography and array stability

Fundus photographs were taken pre-implantation, immediately after implantation and then monthly post-operatively. These images were used to determine the stability of the implant. For this, in each image the boundary of the array, the optic nerve head and retinal vessels were traced using computer-aided-design software (Autodesk) and then aligned/stretched to match the vasculature of the baseline fundus image [26].

#### Optical Coherence Tomography

Single-line high-resolution scans were taken at various time points across the tip of the implant (closest to the optic disc), in the middle of the implant body, and immediately adjacent to the array. OCT was performed before, during and at the end of the chronic stimulation period. Each scan was averaged from 100 frames. The retinal thicknesses of the three locations were measured using the measurement tools within the HEYEX viewing software, which is available in the Spectralis OCT system. At each array location, two tissue thicknesses were determined: (1) total retinal thickness (taken from the ‘retinal pigment epithelium – tapetum junction’ to the ‘retinal nerve fibre layer – vitreous junction’); and (2) the thickness of the retina plus choroid (including the tapetum) to investigate potential choroidal swelling or shrinkage following electrical stimulation.

#### Electroretinography

Recording of the full-field ERG was performed using an Espion E2 system (Diagnosys LLC, Lowell, MA, USA) after 20 minutes of dark adaptation. Both the implanted and the fellow non-implanted control eye were recorded simultaneously. The retinal responses to various light flash luminance levels (0.01–10 cd.m^−2^) were recorded, however, only the combined rod-cone maximal ERG response (10 cd.m^−2^) was reported here as this ERG response provides information on the functional integrity of both the outer retina photoreceptors (a-wave) and mid retina bipolar cells (b-wave) [35]. The ERG responses before and after chronic electrical stimulation were compared.

#### Micro-focus X-ray and fluoroscopy

A custom built micro-focus X-ray imaging system was used in this study [36]. The system featured a micro-focus X-ray source (<10 µm focal spot diameter), a small aperture, a large object-image distance, a high-resolution X-ray detector, and produced a high resolution magnified image. The anaesthetised subject was placed in the X-ray chamber with the implanted eye above the X-ray source. The subject’s head was tilted down, with a line joining the ear-canal and nostril making a 45 degree angle to the horizontal. Still images were taken on an imaging plate (CR-MD4.0; AGFA) with the exposure setting of 55 kV; 1 mA; 3 seconds, to identify the electrode position in the eye and verify the integrity of the implant. The position of the electrode insertion as well as the intra-orbital lead’s freedom of movement were observed via micro-focus fluoroscopy (exposure setting: 55 kV, 50 µA) in real time. The dynamic fluoroscopic image was recorded. A fine-toothed forceps was used to perform forced-duction during fluoroscopic imaging. Imaging was performed after the chronic stimulation period, prior to the final electrophysiology session.

### Electrically-evoked Potential Recordings

Immediately prior to the commencement of chronic stimulation, and monthly thereafter, subjects were anaesthetised for electrophysiological monitoring. Each electrode was stimulated with biphasic cathodic-first 400 µs pulses with currents from 0 to 1.8 mA (maximum charge density <255 µC.cm^−2^ per phase; pp), in steps of 100 µA, at a rate of one pulse per second in monopolar configuration using a custom-designed laboratory stimulator. Current levels were delivered in a random order, and each current level was averaged over 10 repetitions to produce an input-output function, and the whole process was then repeated [37]. The implanted extradural electrodes located in the skull overlying the visual cortex were used to record electrically-evoked visual cortex potentials (eEVCPs) with reference to the implanted stainless steel reference electrode (recording amplifier gain: 100×; sampling rate for analogue-to-digital converter: 100 kHz; bandpass filtered from 10 Hz–2 kHz; 500 ms recording duration per pulse). eEVCPs were analysed in detail offline and the threshold was established for each electrode. The threshold for each response was defined as the lowest current that yielded positive-going peaks, within 30 ms of stimulus onset, of at least 300 µV [37].

### Chronic Stimulation

Chronic stimulation began after subjects recovered from surgery and the eEVCP thresholds for each electrode in the implanted array had been measured. Whilst the subject was awake, the wearable stimulator was activated. All chronic electrical stimulation was performed using charge balanced, biphasic, current pulses at a maximum rate of 200 Hz (Table 3), using monopolar stimulation, with electrode shorting and capacitive coupling to ensure complete charge recovery [38]. The stimulation pulses were presented to the active electrodes on the array in a continuous random sequence. On the first day, stimulation commenced at sub-eEVCP-threshold levels and the current level was incrementally increased until a repeatable perceptual response (a distinct attending motion of the head) was noted. The stimulus current level was then gradually increased (by approximately 10 µA/day) for 10–14 days until either the stimulator reached its voltage compliance limit or the subject responded adversely (i.e. a behavioural response consistent with a noxious stimulus). The maximum stimulation level was then reduced to a level within compliance/tolerance levels and maintained for the duration of the implantation period. Stimulators were powered by two Zinc-Air cells (PR44/p675; 1.45 V each; Cochlear), which were changed every 2 days. Before and during the stimulation period, electrode impedances were measured regularly (see above) and if an electrode was found to be faulty, it was excluded from the stimulation protocol. As such, a proportion of electrodes on each array were not “maximally” stimulated (i.e. not stimulated as much as it could have been if there were no faults). Table 3 summarises the number of electrodes in each array that were stimulated maximally and partially, as well as the number of electrodes in each array that were not stimulated at all. During chronic stimulation, all subjects were housed individually with free access to food, water, bedding and chew toys. Subjects were exercised and monitored daily.

### Acute Electrophysiology

#### Electrically-evoked visual cortex potential recordings

A terminal 48–72 hour electrophysiology experiment was performed at the completion of each subject’s chronic stimulation period, in a similar manner to previously published acute implantation studies [37], [39], [40]. Subjects were anaesthetised as described above and secured in a stereotaxic frame. The final set of eEVCP recordings was performed according to the above protocol. Briefly, a craniotomy was performed above the right visual cortex (i.e. contralateral to the implanted eye). The eEVCP was measured with a Pt ball (1.5 mm diameter) placed on the dura referenced to a subcutaneous Pt needle, at the nape of the subject’s neck. Stimulating with all suprachoroidal electrodes ganged together, the Pt ball was moved rostrocaudally along the dura until the site of lowest threshold was localised.

#### Electrically evoked multi-channel, multi-unit recordings from visual cortex

The dura was excised over the area of visual cortex with the lowest threshold eEVCP and a 60 channel (6×10) penetrating electrode array (with 1 mm long electrodes spaced at 400 µm) was pneumatically inserted (Blackrock Microsystems, UT, USA). Each suprachoroidal electrode was stimulated in a randomised order with 400 µs cathodic-first pulses at defined current levels (10 repetitions at each current level). Multi-unit cortical action potentials were recorded from each recording channel and a digital trigger activated a time-stamp whenever the signal exceeded 4.2 times the root mean square of the background noise. The number and timing of triggered action-potentials were analysed in a window 3 to 20 ms post-stimulus onset and plotted versus each current level. A sigmoid curve was fitted to this current vs. firing rate plot and the current level required to elicit 50% of the maximum firing rate was defined as the threshold [31]. A histogram of the timing of triggered action potentials post-stimulus was created using 1 ms bin widths. For each subject, the lowest cortical multi-unit threshold to stimulation of any retinal electrode was ascertained.

### Termination and Tissue Preparation

The complete procedure for terminating subjects and preparing the eye tissue for analysis has been described previously [32]. Briefly, subjects were overdosed with barbiturate (Pentobarbitone, 150 mg/kg, Ilium, Troy Laboratories pty ltd, Australia), intra-venously (i.v.) heparinised (DBL Heparin Sodium injection BP; 0.1%; Hospira Aust Pty Ltd, Victoria Australia) and transcardially perfused with warm (37°C) saline and then cold (4°C) neutral buffered formalin (NBF). The termination and perfusion procedure followed a consistent and standard protocol for each subject. Eye globes were removed together with implanted electrode arrays and leads. The enucleated globes were post-fixed in Davidson’s fixative before being dehydrated in ethanol. Scleral tissue adjacent to each individual electrode location was identified and dyed (colour-coded) along with the matched position in the contralateral control eye. The eyes were dissected and full thickness tissue strips were collected to include all electrode-adjacent sites. The equivalent “mirror-matched” strips were also collected from the contralateral control eye. Tissue strips were processed and sectioned according to standard histological procedures [32].

### Histological and Immunohistochemical Analyses

Complete details for standard hematoxylin and eosin (H&E) and specific histochemical staining as well as immunohistochemical visualisations have been described previously [32]. Briefly, 5 µm thick paraffin embedded serial sections were collected from each of the dyed tissue regions and mounted on slides. H&E and specific staining, including periodic acid-Schiff (PAS), Gram and Perl’s Prussian blue was performed to identify fungi, bacteria and site/severity of haemorrhage, respectively. Sample sites which were poorly stained, displayed artifact, or were folded or torn on the slide were excluded from subsequent analysis [41]. H&E staining was performed routinely on serial sections, whilst specific staining was performed on random representative samples to rule out any other significant pathology. All sections were imaged using an Axio Imager 2 upright microscope (Carl Zeiss) and Axio Vision software (version 4.8.2; Carl Zeiss). Confocal microscopy examination after immunohistochemical staining was also performed on random representative samples to rule out any other significant glial or neuronal changes. They included anti-glial fibrillary acidic protein (GFAP; AB5804 anti-rabbit polyclonal, Millipore), anti-neurofilament (NF-200; N0142 monoclonal anti-mouse, Sigma-Aldrich) and anti-glutamine synthetase (GS; MAB302 monoclonal anti-mouse, Millipore). These stains were used to, respectively, assess the proliferation of glial cells that occurs in response to neural injury, examine the structure of ganglion cell neurites within the retina and highlight the growth of Müller supporting cells through the retinal layers. DAPI is a nuclear counterstain that was used to highlight the underlying cellular architecture. Immunohistochemical staining was imaged using Nikon eclipse Ti Confocal microscope (Nikon) and NIS-Elements (version 4.10) software. One representative H&E stained section, without artifactual cutting damage and with clear dye markings, was chosen at each electrode-adjacent site as well as the matched location from the contralateral control eyes. These samples (n = 169; out of a possible 196 - if all 14 electrode-adjacent sites and their matched control pairs were retrieved, for all 7 subjects that received stimulation, after sectioning) were used for both retina and fibrosis measurements as well as pathology assessments and scoring.

### Retina and Fibrosis Measurements

**Figure 2:**
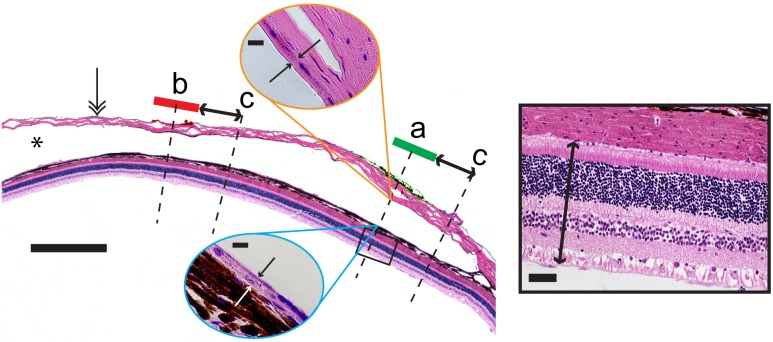
Method for measuring retinal and fibrous thickness. Low power micrograph illustrating the histology following chronic implantation and electrical stimulation of a suprachoroidal electrode array. The sclera (double arrowhead) and the space occupied by the electrode array (*) are clearly visible. Note that the electrode array is removed prior to histology. Retinal thickness was measured adjacent to each electrode – as located by the presence of tissue dye (“a” and “b”), as well as approximately 500 µm distal to each electrode (“c”; in a randomly selected direction). Measurements were made from the ‘retinal pigmented epithelium – tapetum junction’ to the ‘retinal nerve fibre layer – vitreous junction’ (inclusive; black boxed inset). Similarly, fibrous thickness was measured on the choroidal and the scleral sides of the implant in the same locations as the retinal thickness measurements (blue and orange oval insets for choroidal and scleral locations, respectively). Control measurements were made for retinal thickness in a paired location on the contralateral control eye. Finally, implanted eye measurements of retinal thickness and fibrosis were correlated *post-hoc* with lab records of which electrodes were chronically stimulated and which were not [32]. Main panel scale bar = 1000 µm. Solid box scale bar = 500 µm. Blue and orange oval inset scale bars = 10 µm.

Retina and fibrous thickness measurements were made from high power digitised photomicrographs (Axio Imager 2 upright microscope; Carl Zeiss) using Axio Vision measurement software (version 4.8.2). Retinal thickness was measured from the ‘retinal pigment epithelium – tapetum junction’ to the ‘retinal nerve fibre layer – vitreous junction’. Fibrous delineation was defined as a lining of continuous strongly eosin stained acellular collagen between the inner boundary of the space occupied by the electrode array and the choroid, or the lighter eosin stained sclera. Linear measurements were made, taking care to remain as perpendicular to the retina as possible. Measurements were made at a position in the centre of the each dyed scleral region (indicating the former position of an electrode in the array) as well as in the equivalent position in the contralateral control eye. Measurements were also made at a distance (approximately 500 µm) from the edge of the dyed scleral region (Figure 2). All measurements were made by a single operator blinded to the stimulation levels delivered to the electrodes.

### Pathology Assessments and Scoring

There were twelve 600 µm active electrodes and two 2 mm return electrodes in each implanted array (total of 14 Pt electrodes per array). Following processing and paraffin embedding, representative sections (5 µm thick, H&E stained) from each electrode-adjacent tissue sample, and its paired control sample from the non-implanted eye, were assessed by two pathologists independently (following similar procedures to previous studies) [32], [41]. The 169 retrieved samples (see above) were randomised and presented to each pathologist separately in a double-blind fashion. The tissue at each site was scored from ‘0’ (least severe pathology) to ‘4’ (most severe pathology). Scores were assigned for eight metrics of stimulus-induced tissue damage: (1) scar tissue; (2) fibroblastic response; (3) chronic inflammatory response; (4) acute inflammatory response; (5) foreign body multinucleate giant cell response; (6) necrosis; (7) retinal damage; (8) other damage remote to implant. Scores were assigned with knowledge and experience of the normal trajectory of chronic passive implantation [26], and reflected any observed increase from this defined baseline. Any discrepancy in inter-pathologist scores (which occurred in <2% sections) was discussed and a consensus was reached. To verify intra-operator consistency, 10% of samples were covertly retested. After all scores were gathered, the randomisation scheme was decrypted and each electrode-adjacent tissue sample was correlated with records of the chronic stimulus parameters used for that electrode.

### Statistics

Statistical modelling was carried out in Genstat (15^th^ edition); linear mixed model procedures that account for multiple measurements taken on the same subjects were used where necessary. The results are presented as estimated mean differences with 95% confidence intervals, for comparisons of interest. A *p*-value for the comparison is also provided. More details of the analyses are provided with the results.

## Results

### Cohort, Stimulation and Electrode Survival

Table 3 summarises the cohort and electrode survival. Seven subjects were implanted with suprachoroidal electrode arrays for 96–143 days. Of these, six were stimulated continuously for 70–105 days (mean: 88 days; standard deviation: 15 days), and one (subject 505) was only stimulated continuously for 18 days (due to a faulty lead system). All subjects were stimulated using a monopolar electrode configuration – utilising the large intraocular electrodes as the return path. Stimulus intensities were gradually increased (Figure 1) up to maximum levels ranging between 263–541 µA for the six subjects stimulated for approximately 3 months. These current levels corresponded with geometric charge densities of 37–77 µC.cm^−2^ pp for the 600 µm diameter electrodes used in the present study. During the course of the implantation and stimulation period some electrode channels failed and were disconnected from the stimulator. For the 6 subjects stimulated for approximately 3 months each (i.e. excluding subject 505), the number of 600 µm diameter electrodes on each array that received stimulation during the course of the study ranged from 9–12 (out of 12). All electrode channel failures were attributed to the percutaneous lead and/or connector rather than the implant array itself which appeared intact in all post-explantation microscopic analyses. Additionally, a small cohort of electrodes received no stimulation during the study and therefore served as unstimulated controls (Table 3). In the present paper, the combined set of “maximally stimulated” and “partially stimulated” electrodes is collectively referred to as “stimulated” electrodes.

### Surgical Recovery and Overall Health

All subjects recovered from the implantation surgeries within 1–2 days. All subjects returned to pre-surgical weight by 2 weeks post-implantation. Subjects tolerated both the implants and backpack harnesses well with the exception of a minor infection in subject 503 at the surgical wound near the apex of the skull which was managed with topical antibiotics (Betadine antiseptic solution, Sanofi-aventis consumer healthcare, Australia). A gradual increase (ramp up) of stimulation intensity over approximately 2 weeks (Figure 1) gave subjects acclimation time and the maximum stimulation levels were, in all cases, governed by stimulator voltage compliance and not subject intolerance. At no time was there evidence of visible or electrophysiologically recorded myogenic activity associated with stimulation through the electrode array.

### Clinical Assessments

#### Fundus photography and array stability

**Figure 3:**
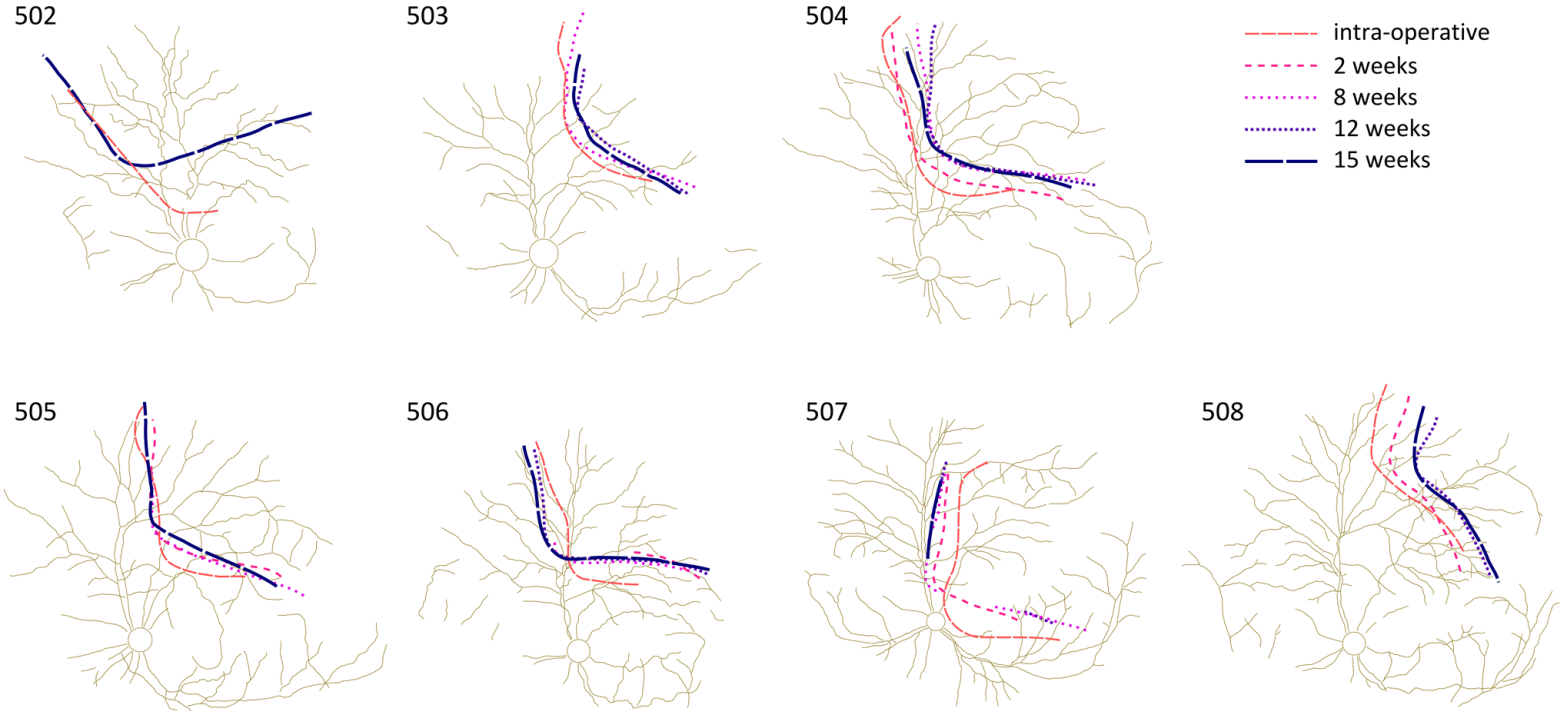
Long-term mechanical stability of the suprachoridal electrode array taken from longitudinal fundus images. Digitised fundus images recorded immediately following surgery, and over the 15 week chronic implantation period, illustrating the mechanical stability of the implant with respect to the optic nerve and retinal vasculature. Each line depicts the edge of the suprachoroidal electrode array visualised through the fundus image (supplementary Figure S1). An initial settling of the implant was observed during the first weeks with a displacement of 1–2 optic disc diameters (optic disc diameter is approximately 1.5 mm) in the implant outline. The implant location remained stable after 8 weeks of implantation. Note that data from all time points were not always available.

The mechanical stability of the electrode array was assessed from fundus images (Figure 3). There was an initial settling period lasting up to 2 weeks, after which the array position did not change substantially with respect to the retinal vasculature.

#### Optical Coherence Tomography

**Figure 4:**
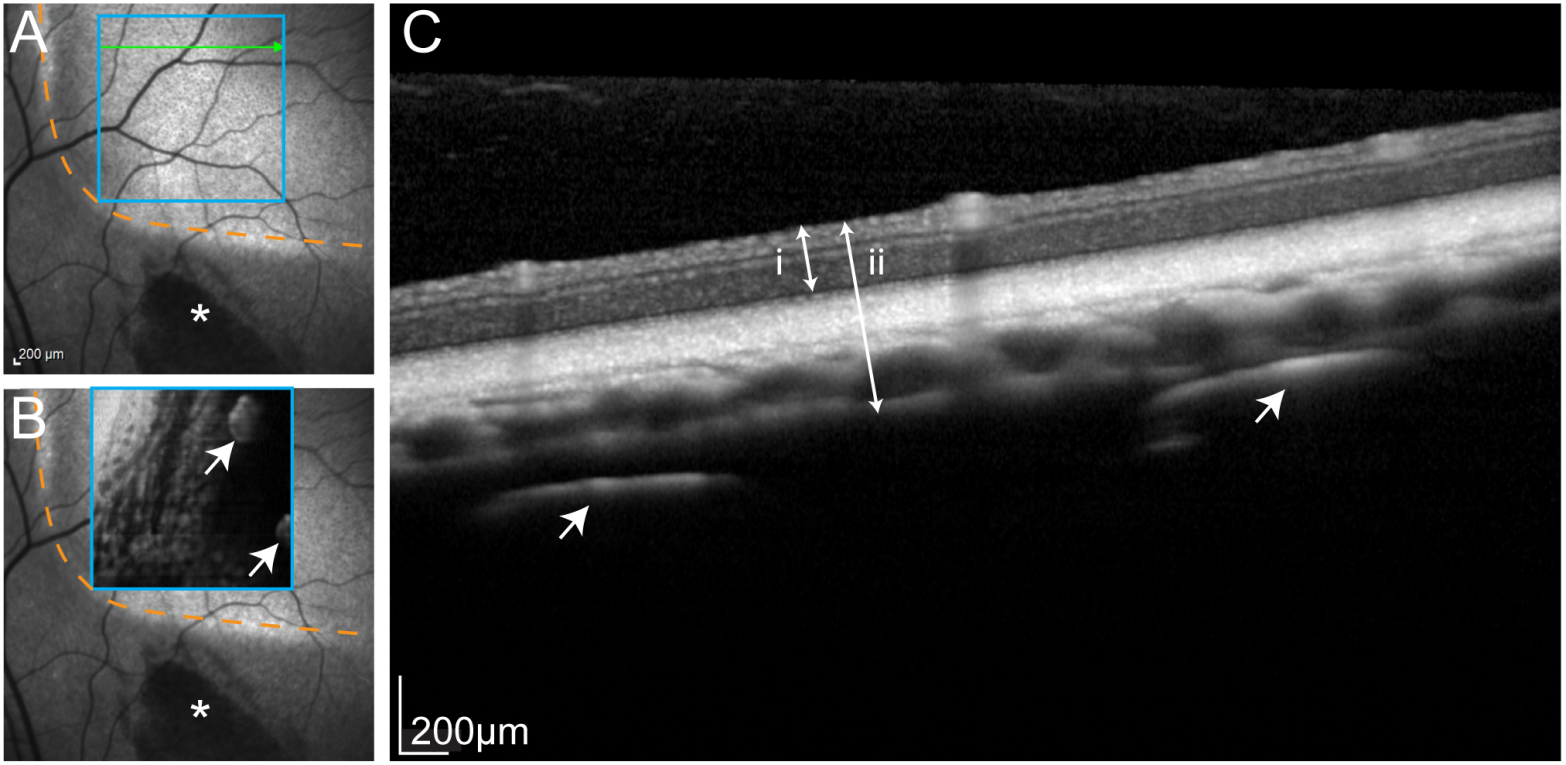
Representative optical coherence tomography. (**A**) Infrared fundus image illustrating the typical location of the electrode array in the superior retina. Orange dashed lines indicates the inferonasal extent of electrode array. Asterisk denotes hyperpigmentation near the tip of array caused by minor insertion trauma (as reported previously [26]). (**B**) Optical coherence tomography (OCT): en face scan of region delineated by blue square, showing individual 600 µm platinum (Pt) electrodes (arrows) within the body of the array. Orange dashed line and asterisk are as described in (A). (**C**) Cross-section through the green line in (A) revealing normal retinal morphology and vasculature. Arrows show two 600 µm Pt disc electrodes. Double-ended arrows show measurements used for determining retinal (i) and ‘retina plus choroid’ thickness (ii). Overall, the OCT scans suggested all subject exhibited healthy eye tissue throughout the course of the study.

Figure 4 shows typical images of an implanted eye after 3 months of implantation and stimulation. The outline of the silicone array body was clearly visible on the infrared reflectance image (Figure 4A) and the individual Pt electrodes within the array were detected on the OCT line scan (Figure 4B & C). OCT images did not reveal any signs of retinal damage, folding or delamination. The retinal architecture, tapetal layer, and choroid all appeared healthy. The optical penetration of the OCT was insufficient to image the sclera behind the array. In some cases, there was hyper-pigmentation near the tip of the electrode array, consistent with previous descriptions of minor implantation trauma and thinning of the tapetal layer [26].

**Figure 5:**
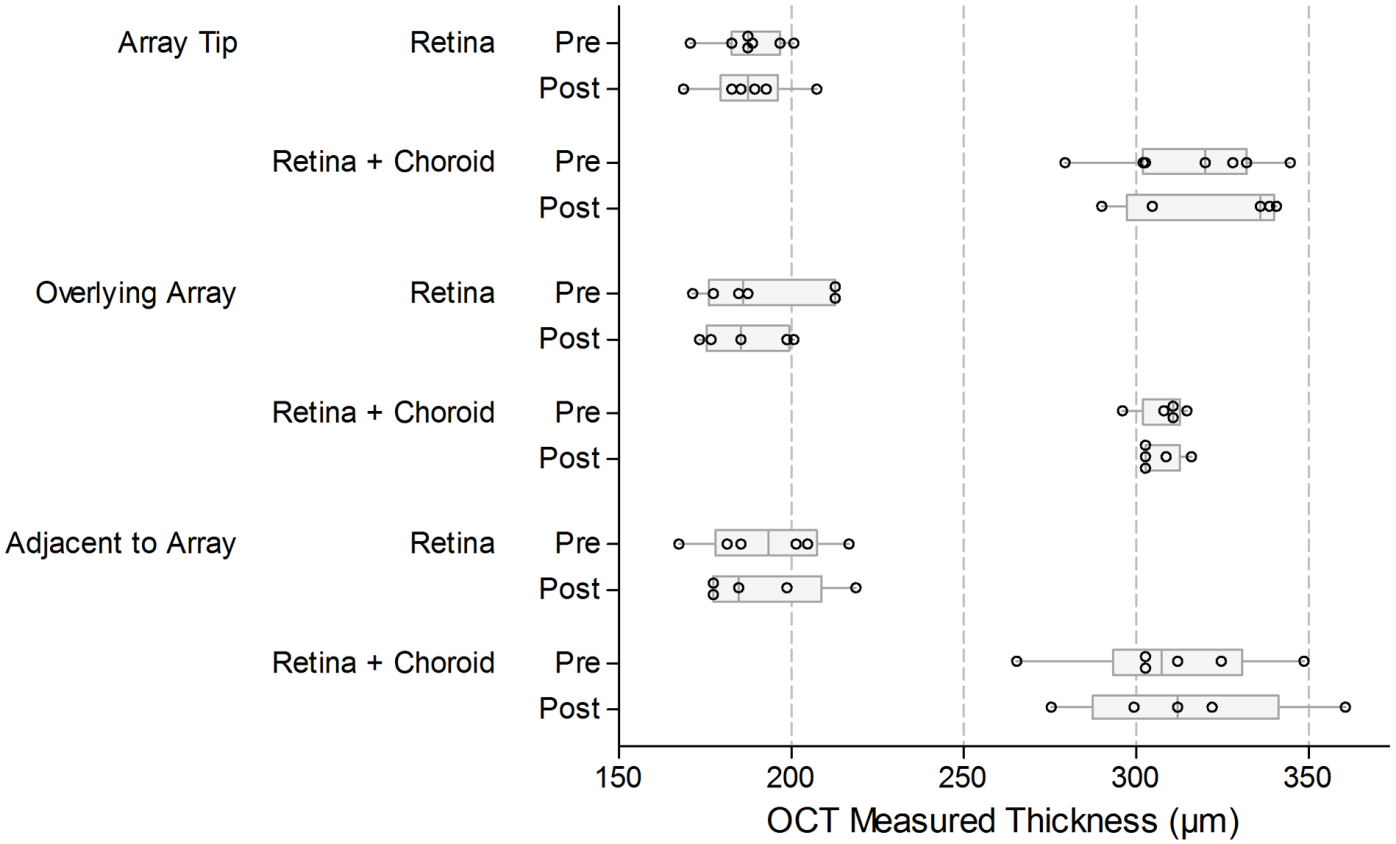
Optical coherence tomography (OCT) measured retinal and choroidal thicknesses before and after chronic electrical stimulation. There was no evidence of a change in either the retinal or retinal plus choroid thickness as a result of the chronic stimulation program either directly underlying or distal to the electrode array. The thickness of the retina, and the retina plus choroid (including the feline tapetal layer) was measured (µm) for each subject (refer to Figure 4). Measurements were made: at the tip of the implanted array, at a location overlying the middle of the array, and at a location directly adjacent to the array (within 500 µm). Measurements were made before the commencement of chronic stimulation (Pre) and at the completion of the chronic stimulation period (Post). Box plots show median (midline), 1^st^ and 3^rd^ quartiles (box edges), whiskers have a maximum length 1.5 times the interquartile range and open circles represent individual points. There are between 5 and 7 data points (subjects) contributing to each boxplot. There was substantial overlap of the pre- and post-stimulation measures for retinal, or retinal plus choroidal, thickness in each of the locations. The estimated mean differences were small, as were the limits of the confidence intervals; none of the comparisons were statistically significant (Table 4).

Across all locations, the average retinal thickness was 187 µm, and the average retina plus choroid (including tapetum) thickness was 308 µm. Boxplots of these OCT measured thicknesses are shown in Figure 5. Table 4 provides estimates of the mean difference in retinal or ‘retinal plus choroidal’ thickness before and after chronic stimulation, based on paired *t* procedures. These mean differences were generally small, and all the *p*-values were large, indicating the comparisons were not statistically significant.

**Table 4:** Mean difference in OCT measured thickness (‘before’ minus ‘after’ chronic stimulation).

		Mean difference in µm (Before – After)		
		Estimate	95% confidence interval	*P*–value
Array tip	Retina	0.00	–6.16, 6.16	>0.995
Array tip	Retina + Choroid	4.80	–4.63, 14.23	0.231
Overlying array	Retina	–1.50	–18.26, 15.26	0.794
Overlying array	Retina + Choroid	–0.20	–11.49, 11.09	0.963
Adjacent to array	Retina	3.50	–2.66, 9.66	0.168
Adjacent to array	Retina + Choroid	–3.75	–17.15, 9.65	0.439

#### Electroretinography

**Figure 6:**
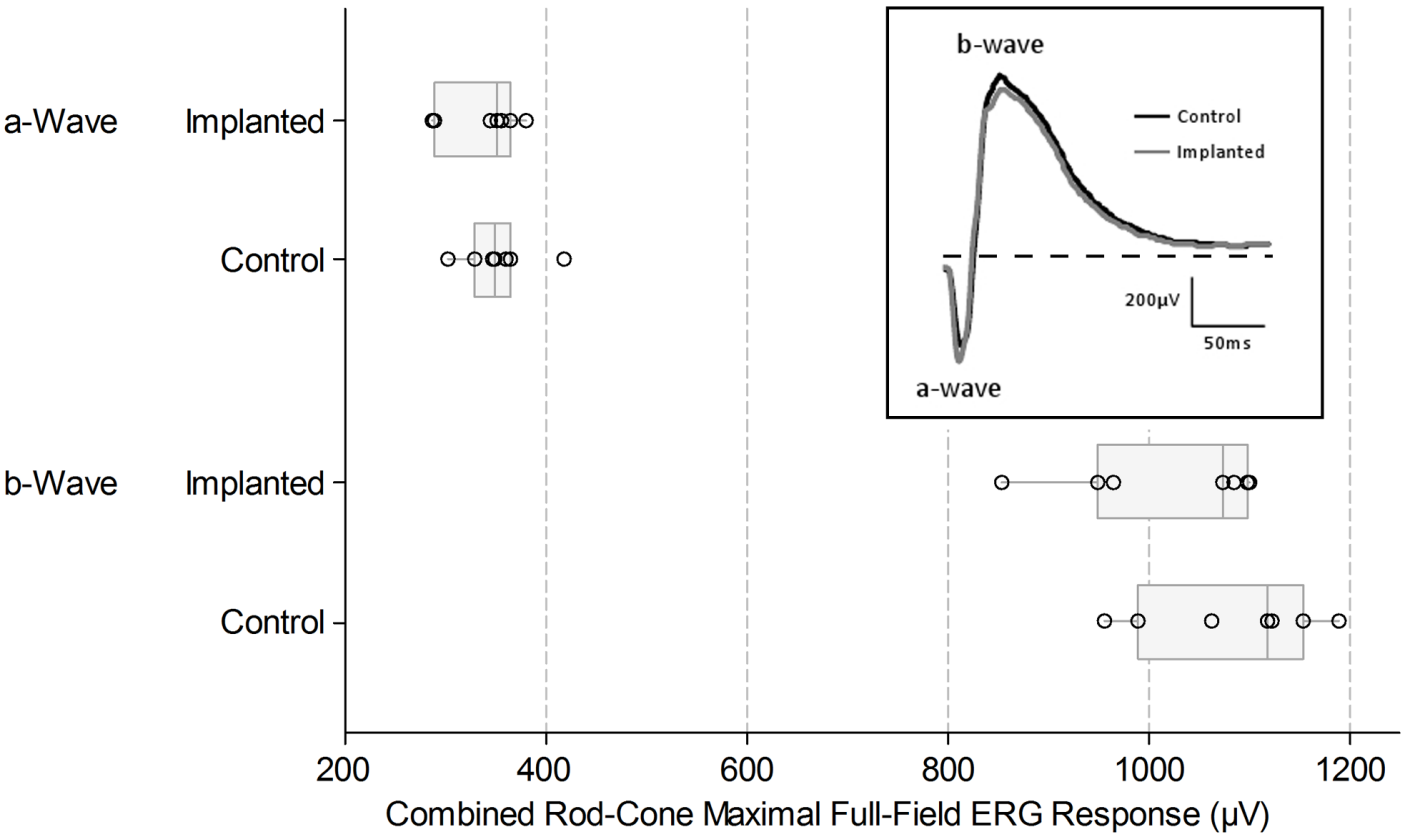
Combined rod-cone maximal full-field electroretinogram (ERG) responses at the completion of the chronic stimulation period. Examples of the ERG waveforms are shown in the inset with the a- and b-waves indicated. Dashed horizontal line indicates recording baseline. a-wave amplitude is taken from baseline to a-wave trough; b-wave amplitude is taken from a-wave trough to b-wave peak. The median and interquartile range of ERG responses from n = 7 subjects are presented in the box plots. Box plots show median (midline), 1^st^ and 3^rd^ quartiles (box edges), whiskers have a maximum length 1.5 times the interquartile range and open circles represent individual points. There was substantial overlap between the measured response amplitude of the a- and b-waves between the implanted and control eyes. The paired *t* procedures showed the mean difference (implanted minus non-implanted) for the a-wave was −14.3 µV (95% confidence interval: –50.5, 21.8, *p* = 0.37), and for the b-wave was −66.6 µV (95% confidence interval: –135.9, 2.8, *p* = 0.057). Minor differences between eyes can be attributed to electrode placement or normal biological variation.

A representative light-activated ERG response at the end of the chronic stimulation is shown in Figure 6 (inset). The paired *t* procedures showed the mean difference (implanted minus non-implanted) for the a-wave was −14.3 µV (95% confidence interval: −50.5, 21.8, *p* = 0.37), and for the b-wave was −66.6 µV (95% confidence interval: −135.9, 2.8, *p* = 0.057). These indicate that there were no notable mean differences in the maximal ERG responses (for neither a- or b-wave) between the implanted and non-implanted control eyes at the end of the chronic stimulation period (Figure 6).

#### X-ray and fluoroscopy

X-ray imagery confirmed the position of the electrode array in the posterior eyeball at the horizontal meridian. The electrodes were all accounted for in their correct arrangement and there was no overt buckling or twisting of the array within the implant cavity. The lead loop moved freely within the extra-ocular space and the helical coil did not kink during forced duction tests. There was minor stretching of the helix at the scleral exit and the orbital margin (Video S1).

### Electrode Impedances

**Figure 7:**
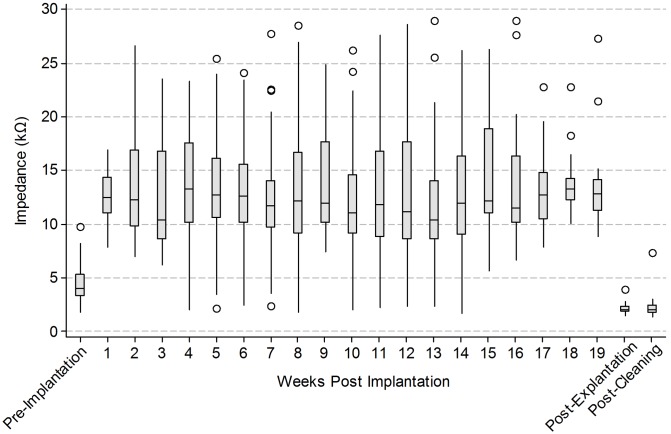
Longitudinal changes in electrode impedance. Electrode impedance (kΩ) across all subjects recorded periodically over the duration of the chronic implant period. Box plots show median (midline), 1^st^ and 3^rd^ quartiles (box edges), whiskers have a maximum length 1.5 times the interquartile range and open circles denote outliers. Pre-implantation, post-explantation and post-cleaning impedance measurements were performed in normal saline. The number of individual electrode measurements that comprise each box plot ranged from 21 to 67 (maximum possible = 72; subject 505 was excluded due to a damaged lead).

Electrode impedance data from the 6 subjects stimulated for approximately 3 months are presented as boxplots over time (Figure 7). During the implantation and stimulation period, the measured impedances remained stable. Mean impedances typically ranged between 11–15 kΩ. *In vivo* impedances were approximately three-fold higher than pre-implantation levels measured in saline (approximately 4 kΩ). Post-explantation impedances, again measured in saline, were approximately half pre-implantation levels. Cleaning the arrays did not significantly alter the mean impedance. The estimated mean difference between post-explantation and post-cleaning was 0.19 kΩ (95% confidence interval: −0.26, 0.65; *p* = 0.39).

### Electrically-evoked Visual Cortex Potentials

**Figure 8:**
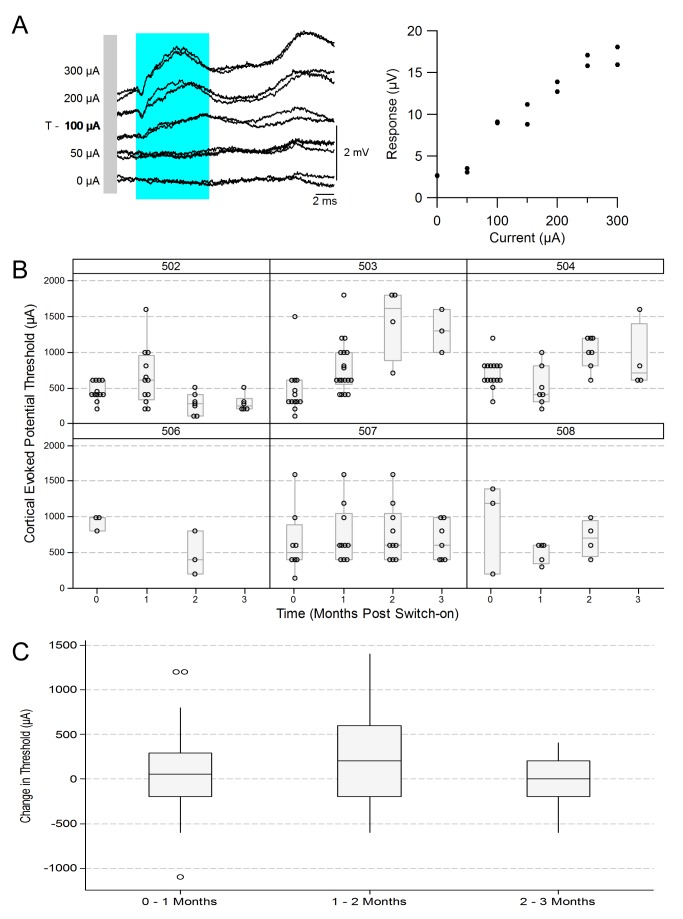
Longitudinal stability of electrically-evoked visual cortex potentials (eEVCP). (**A**) Average cortical responses to increasing current are shown on the left. The stimulus is repeated twice at each current level and both response traces are displayed. The amplitude (mV) of the evoked response (within the region indicated by the blue shaded region) as a function of current (mA) is plotted on the right. In this case, evoked cortical potential threshold was 100 µA (as denoted by “T”). (**B**) eEVCP thresholds for each subject, recorded monthly, starting immediately before the initiation of chronic stimulation (0 months). Box plots show median (midline), 1^st^ and 3^rd^ quartiles (box edges), whiskers have a maximum length 1.5 times the interquartile range and open circles represent individual points. No eEVCP data was available for subject 505 due to a damaged lead. (**C**) The change in threshold, on a per-electrode basis. The changes were calculated for each electrode separately, in monthly increments, and these data were combined. Box plots show median (midline), 1^st^ and 3^rd^ quartiles (box edges), whiskers have a maximum length 1.5 times the interquartile range and open circles denote outliers. There was little variation in median change in threshold over the three time points. The 95% confidence interval comparing switch on to three months was −119.5 to 383.9 µA; this shows a range of plausible values for the overall change over time, including zero.

Electrically-evoked potentials from the visual cortex (eEVCP) were recorded at monthly intervals during the chronic stimulation period (Figure 8). Figure 8A (left panel) shows an example of cortical activity in response to increasing current levels of suprachoroidal stimulation. The right panel of Figure 8A shows the response amplitude within the blue shaded region (left panel) as a function of stimulus current level. eEVCP amplitude increased monotonically from threshold to saturation. The threshold for cortical activation in this example was 100 µA.

Figure 8B shows the eEVCP thresholds recorded monthly in each subject throughout the chronic stimulation period (with the exception of subject 505, which was discontinued before 1 month of stimulation). On a case by case basis, cortical thresholds appear to be relatively stable, with the exception of subject 503 which displayed increasing thresholds during the 3 month stimulation period.

To assess the overall change in cortical threshold over time, the differences in thresholds were combined on a per electrode basis (Figure 8C). There was little variation in the combined median change in threshold over the duration of the chronic stimulation period. A linear mixed model provided estimates of the mean differences in eEVCP thresholds in adjacent months; the largest mean difference was 140.2 µA between the first and second months (Table 5). It also shows the 95% confidence interval comparing switch on to three months as −119.5 to 383.9 µA; this gives a range of plausible values for the change over time, which includes zero (i.e. no change).

**Table 5:** Mean differences in eEVCP thresholds in adjacent months and overall.

*Overall Wald test from Linear mixed model: F(3, 104) = 1.83, P = 0.147*			
	Mean difference in µA		
	Estimate	95% confidence interval	*P*–value
One month – Switch on	67.5	−85.8, 220.8	0.385
Two months – One month	140.2	−42.9, 323.3	0.132
Three months – Two months	−75.5	−347.0, 196.0	0.582
Three months - Switch on	132.2	−119.5, 383.9	0.340

### Electrically Evoked Multi-channel Multi-unit Recordings from Visual Cortex

**Figure 9:**
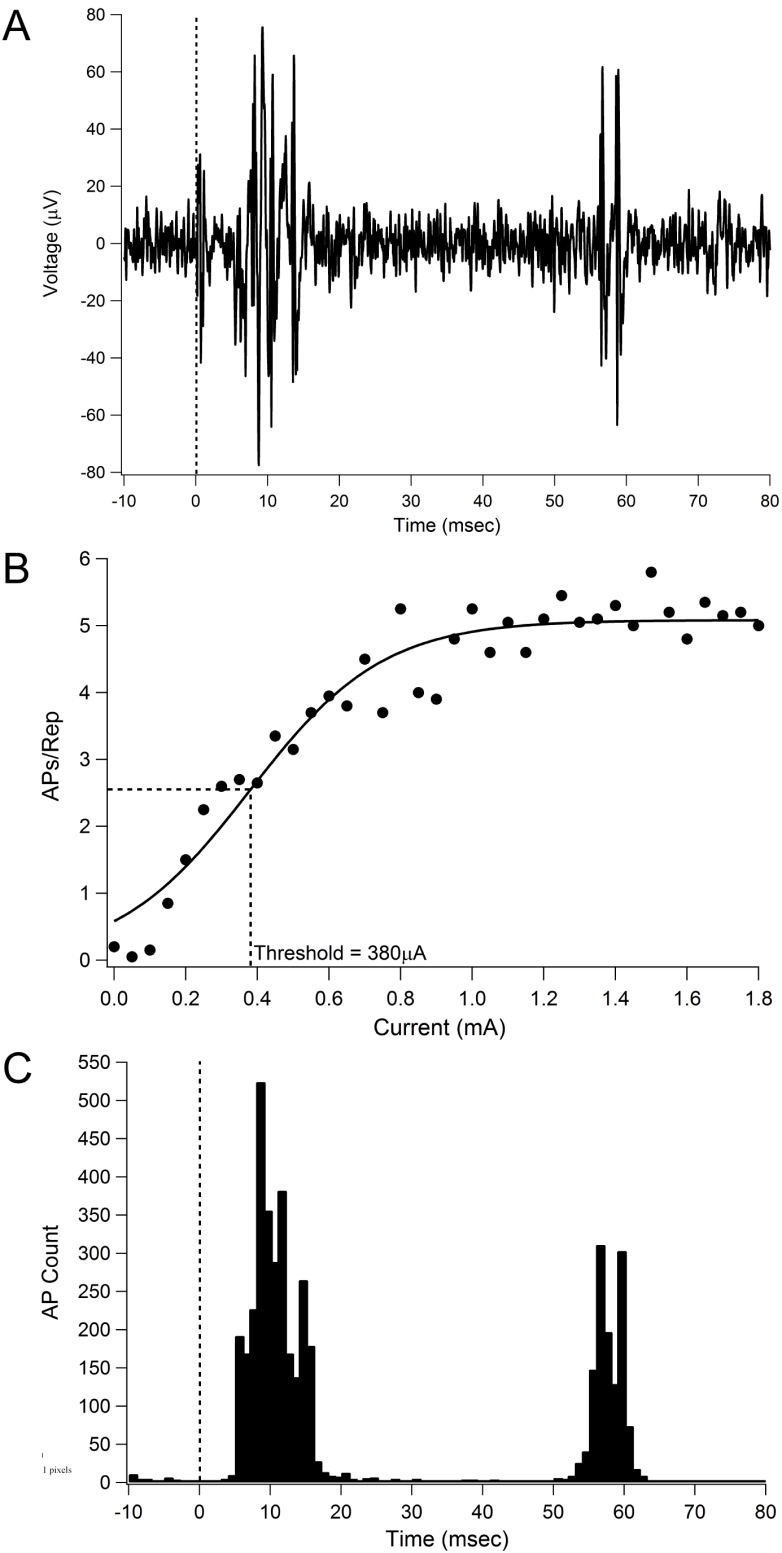
Example Spike Recordings and Multi-Unit thresholds. (**A**) Typical multiunit recording from one cortical channel in response to stimulation of a single suprachoroidal electrode. (**B**) Input-output function from the same cortical channel. Threshold was defined as the current required to elicit 50% of the maximum spike rate. (**C**) Peri-stimulus time histogram (1 ms bin width) across multiple repetitions of all currents presented, indicating two waves of spiking activity post stimulation. Dashed lines in (A) and (C) indicate stimulus onset. Abbreviation: AP, action potential.

Attempts to record multi-unit cortical activity were made in all 6 operational subjects (Figure 9; subject 505 could not be used due to a faulty lead). Evoked-activity was not recorded in 2 subjects, possibly due to a retinotopic mismatch between the suprachoroidal and cortical arrays. In the remaining 4 subjects, the lowest cortical multi-unit thresholds from each of the subjects ranged from 171 µA (in subject 502) to 463 µA (in subject 503). These correspond with charge densities ranging from 24 µC.cm^−2^ pp to 65 µC.cm^−2^ pp. In all subjects where cortical multi-unit recordings were obtained, the lowest cortical threshold was lower than the maximum chronic stimulation level set for that subject; i.e. the stimulus parameters used in the chronic study were sufficient to drive neural activity within the central visual pathway.

### Retinal Histopathology and Immunohistochemistry

#### H & E stained sections

**Figure 10:**
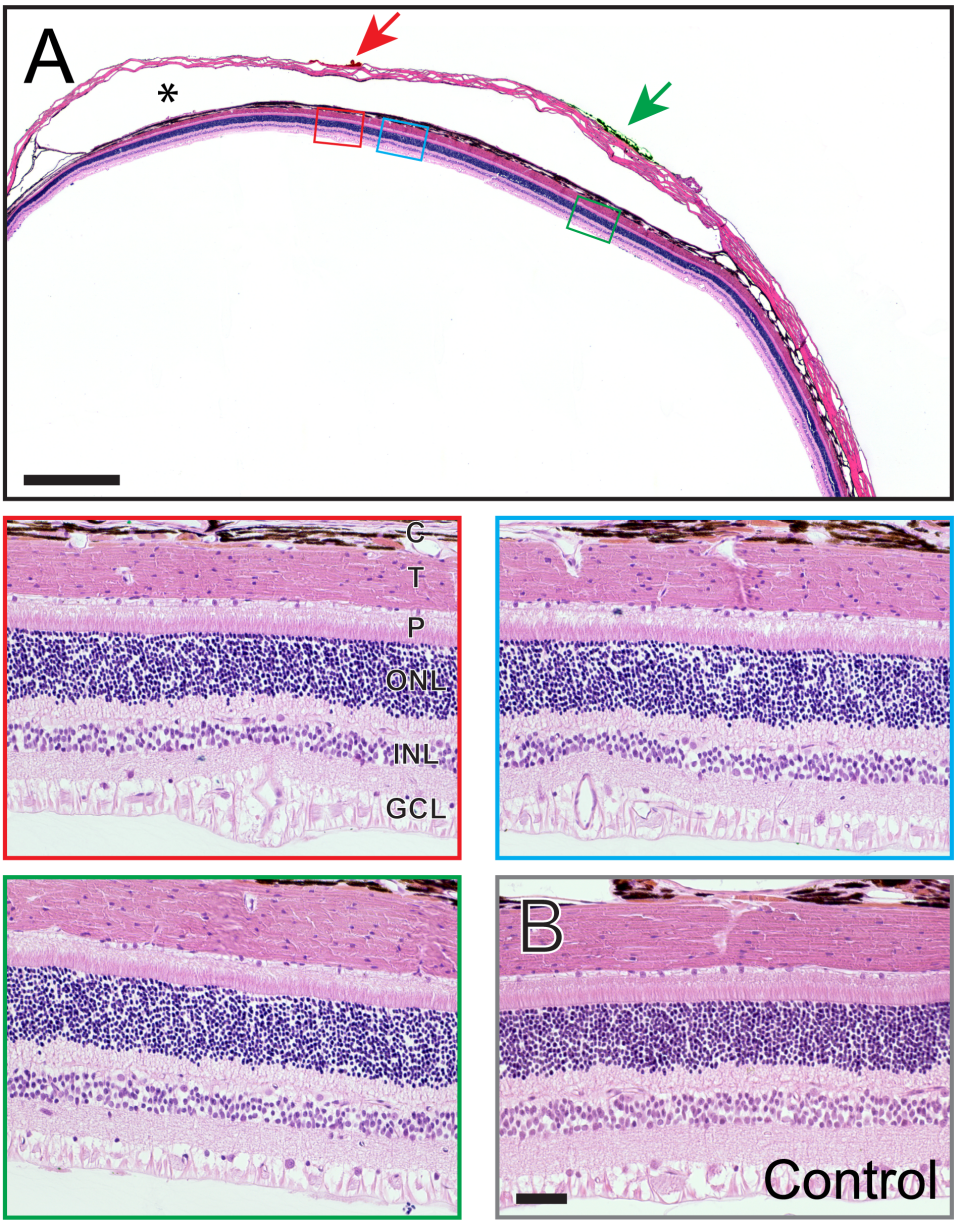
Retinal Histology. (**A**) Photomicrograph of a representative retina following chronic electrical stimulation. The red and green arrows indicate histological dye used to mark the sclera at the site of individual electrodes within the implanted array. In this case, red dye was used to indicate non-stimulated electrodes and green dye to indicate stimulated electrodes. Boxes show magnified tissue regions adjacent to the non-stimulated (red box) and stimulated (green box) platinum (Pt) electrodes as well as 500 µm distal to a Pt electrode (blue box). Scale bar = 1 mm. Asterisk denotes the space occupied by the electrode array. (**B**) Representative example of retina from paired, non-implanted, control eye. The scale bar in panel B = 50 µm, and applies to all magnified boxed regions. In all cases, the inner and outer retina as well as the tapetum, choroid and sclera did not show any significant histomorphological abnormality. There were no observable differences in overall retinal histopathology between samples. Abbreviations: GCL, ganglion cell layer; INL, inner nuclear layer; ONL, outer nuclear layer; P, photoreceptors; T, tapetum, C: choroid. Subject ID: 502.

Figure 10 shows a representative 5 µm retinal section taken through the suprachoroidal implant pocket following 143 days of implantation (98 days of stimulation; subject 502). There was no gross morphological damage to the retina, choroid or sclera observed around the space occupied by the electrode array. The retina was not delaminated and there were no indication of widespread trauma. In this subject, red dye was used to mark sclera adjacent to unstimulated electrodes, whilst green dye was used to mark maximally stimulated electrodes. High power fields of red and green dye-adjacent retinal regions (respectively, red and green boxed insets) as well as a high power field of the retina 500 µm distal to a Pt electrode (blue boxed inset) are shown. In all cases there was healthy retina with no evidence of ongoing foreign body reaction or acute/chronic inflammatory response.

#### Retinal and fibrous thickness measurements

**Figure 11:**
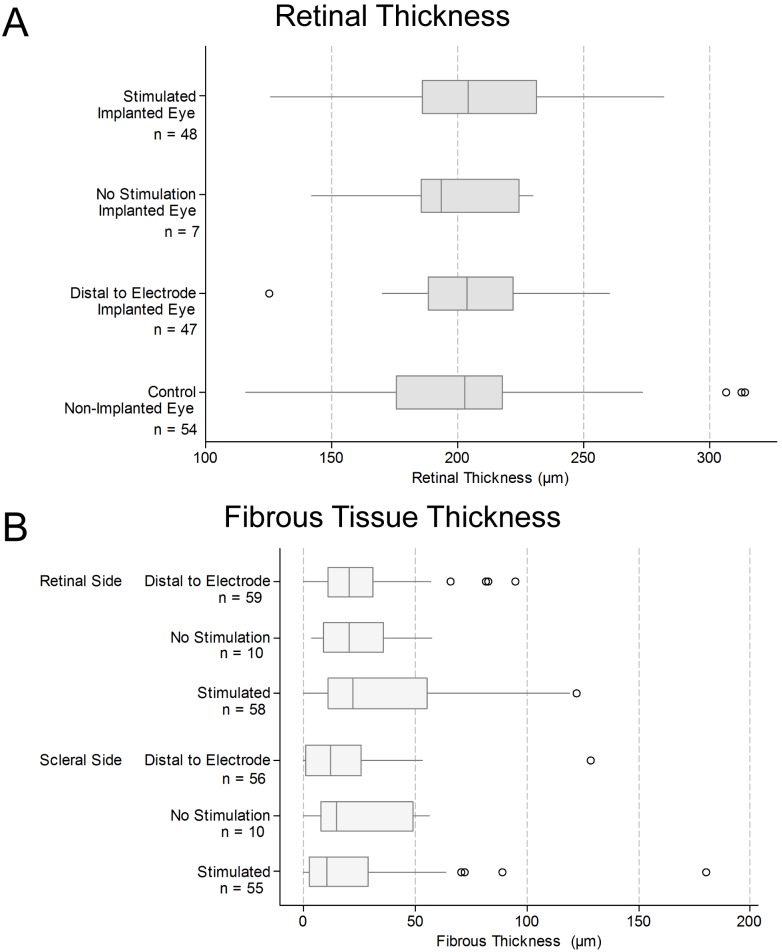
Retina and fibrous tissue thickness measured from histological sections. (**A**) Retinal thickness (µm) was measured in retina adjacent to individual platinum stimulated electrodes, non-stimulated control electrodes, regions approximately 500 µm distal to each electrode (refer to Figure 2), as well as in matched sites from the non-implanted contralateral control eye. There was little variation in the median retinal thickness between measured regions. However, when a linear mixed model accounting for the pairing of observations was used to estimate mean differences in retinal thickness for each condition, a statistically significant difference between stimulated and control conditions was observed. This small estimated mean increase in stimulated retinal thickness (Table 6) was not regarded as being clinically relevant. (**B**) Fibrosis tissue thickness measurements (µm) were made in retina adjacent to stimulated electrodes, non-stimulated electrodes and in a region approximately 500 µm distal to each electrode. Measurements were repeated for the scleral and choroidal sides of the space occupied by the array. There was no fibrosis in the contralateral eye. In both panels, box plots show median (midline), 1^st^ and 3^rd^ quartiles (box edges), whiskers have a maximum length 1.5 times the interquartile range and open circles denote outliers. There was substantial overlap between the fibrosis distributions in the different locations measured, and between the 95% confidence intervals for the mean fibrosis thickness in each condition (Table 7). Note that the sample sizes reflect the number of unique histological measurements performed for each group. Typically, a single measurement is made at each sample or sample-adjacent site. However, in some cases, due to artifactual damage (or because the histology wasn’t retrieved), a particular site was not measured. As a result, the no-stimulation sample size was n = 7 for panel A, and n = 10 for panel B. Both of these samples are a subset of the maximum possible (i.e. n = 14) if all the samples had been retrieved with no histological artifacts.

Figure 11A shows the distribution of measured retinal thickness adjacent to stimulated electrodes, unstimulated electrodes, and 500 µm distal to electrodes, alongside the retinal thickness measured in the control eye (at a mirrored location equivalent to the position of the electrode array in the implanted eye) [32]. The median retinal thickness, measured from the ‘retinal pigment epithelium – tapetum junction’ to the ‘retinal nerve fibre layer – vitreous junction’ was approximately 200 µm. There was overlap in the measured thickness distributions from the different locations assessed. A linear mixed model was used to estimate mean differences in retinal thickness for each condition, compared with the control (Table 6). The mean differences observed were small, and although statistically significant in one case, were not regarded as being clinically relevant as there were no clinically observable changes in ophthalmic structure or function, and no observable glial reaction. Similarly, there were no notable differences between the measurements of the ‘retina plus choroid’ thicknesses, taken in the same locations as the retinal thickness measurements (data not shown).

**Table 6:** Mean difference in histologically measured retinal thickness (comparisons with non-implanted eye).

*Overall Wald test from Linear mixed model: F(3, 96) = 2.75, P = 0.047*			
	Mean difference in µm		
Comparisons with the control	Estimate	95% confidence interval	*P*–value
Stimulated – Control	11.68	3.36, 20.00	0.006
No Stimulation – Control	−2.21	−19.84, 15.42	0.804
Distal – Control	5.84	−2.48, 14.15	0.167

Figure 11B shows the results of the fibrosis encapsulation thickness measurements, from both the choroidal and scleral sides of the implanted electrode array. Measurements were made adjacent to stimulated electrodes, unstimulated electrodes, and 500 µm distal to electrodes. No control eye measurements were used for this assessment as there was no fibrosis in the non-implanted eye. There was minimal fibrosis observed, with median thickness ranging from 15–20 µm, which is consistent with passive implantation results [26]. There was substantial overlap between the boxplots of fibrous thickness from the different locations measured. Table 7 provides estimates of the mean fibrosis encapsulation thickness in each condition in each location; the confidence intervals for the unstimulated electrodes are relatively wider as there were only a small number of measurements made for unstimulated electrodes. The estimates of the means for the stimulation and adjacent measurements are similar, in both the retinal and scleral side. There is also substantial overlap of the confidence intervals for the stimulated and unstimulated tissue regions on both sides of the array.

**Table 7:** Mean fibrosis encapsulation thickness (µm) on retinal and scleral sides of the space occupied by the electrode array (with 95% confidence intervals).

	Mean in µm	
	Estimate	95% confidence interval
Retinal side		
Stimulated	31.91	14.80, 49.02
No Stimulation	24.82	2.05, 47.59
Distal to electrode	24.05	14.59, 33.51
Scleral side		
Stimulated	23.95	6.04, 41.86
No Stimulation	10.00	8.07, 37.70
Distal to electrode	18.42	8.20, 28.64

*Note that separate estimates are provided for each condition, rather than pairwise comparisons of conditions; this is because of difficulty in fitting the statistical models that allow a direct comparison of conditions.*

#### Specific stains

**Figure 12:**
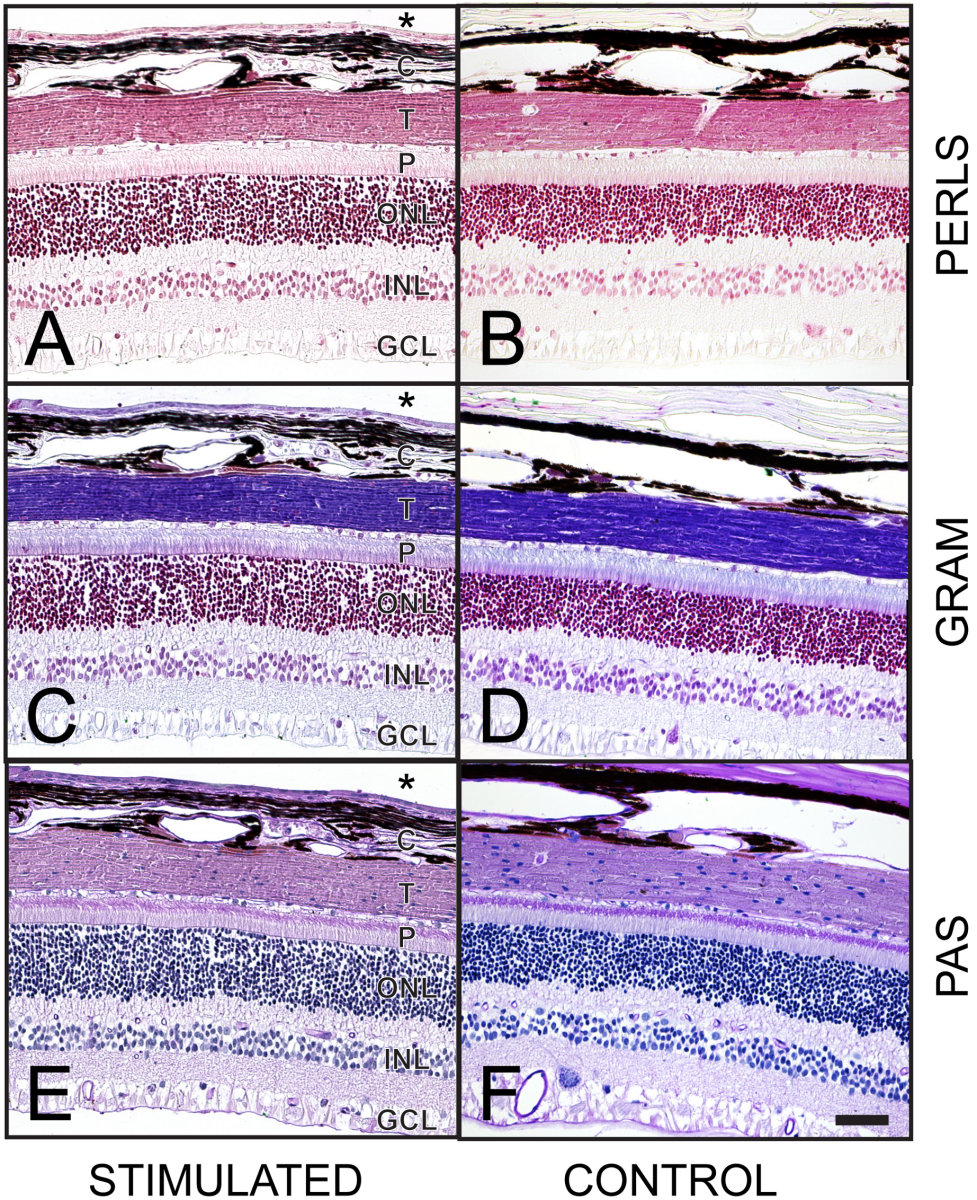
Representative examples of specific histological staining from both stimulated and control eyes. (**A, B**) Perls’ Prussian blue; at the site of haemorrhage, the formation of haemosiderin from degraded red blood cells and release of iron complexes would produce a purple color while the addition of neutral red stain colors lysosomes red. No haemorrhage was noted in any of the samples. (**C, D**) Gram; Gram stain can be used to detect evidence of bacterial infection. None was observed in any of the samples. (**E, F**) Periodic acid-Schiff (PAS); glycoprotein components of basement membrane and connective tissue components are stained purple, while the haematoxylin counterstains cell nuclei purple. PAS stain can be used to detect evidence of fungal infection. None was observed in any of the samples. Scale bar in panel F = 50 µm, and applies to all panels. The inner retina is shown at the bottom of each image; the outer retina is shown at the top of each image. Asterisks denote the space occupied by the electrode array. Abbreviations: GCL, ganglion cell layer; INL, inner nuclear layer; ONL, outer nuclear layer; P, photoreceptors; T, tapetum, C: choroid. Subject ID: 502.

Representative samples from each subject were assessed for additional markers of injury or infection. One stimulated and one control sample from each subject was examined for each of the three specific stains, with the exception of subject 503, for which four stimulated samples were examined for Perl’s stain. Figure 12 shows representative stimulated and unstimulated (non-implanted control) cases stained with PAS, Gram and Perl’s stain. There was no evidence of fungal or bacterial infection, or former haemorrhage in any of the sections examined (n = 45).

#### Immunohistochemistry

**Figure 13:**
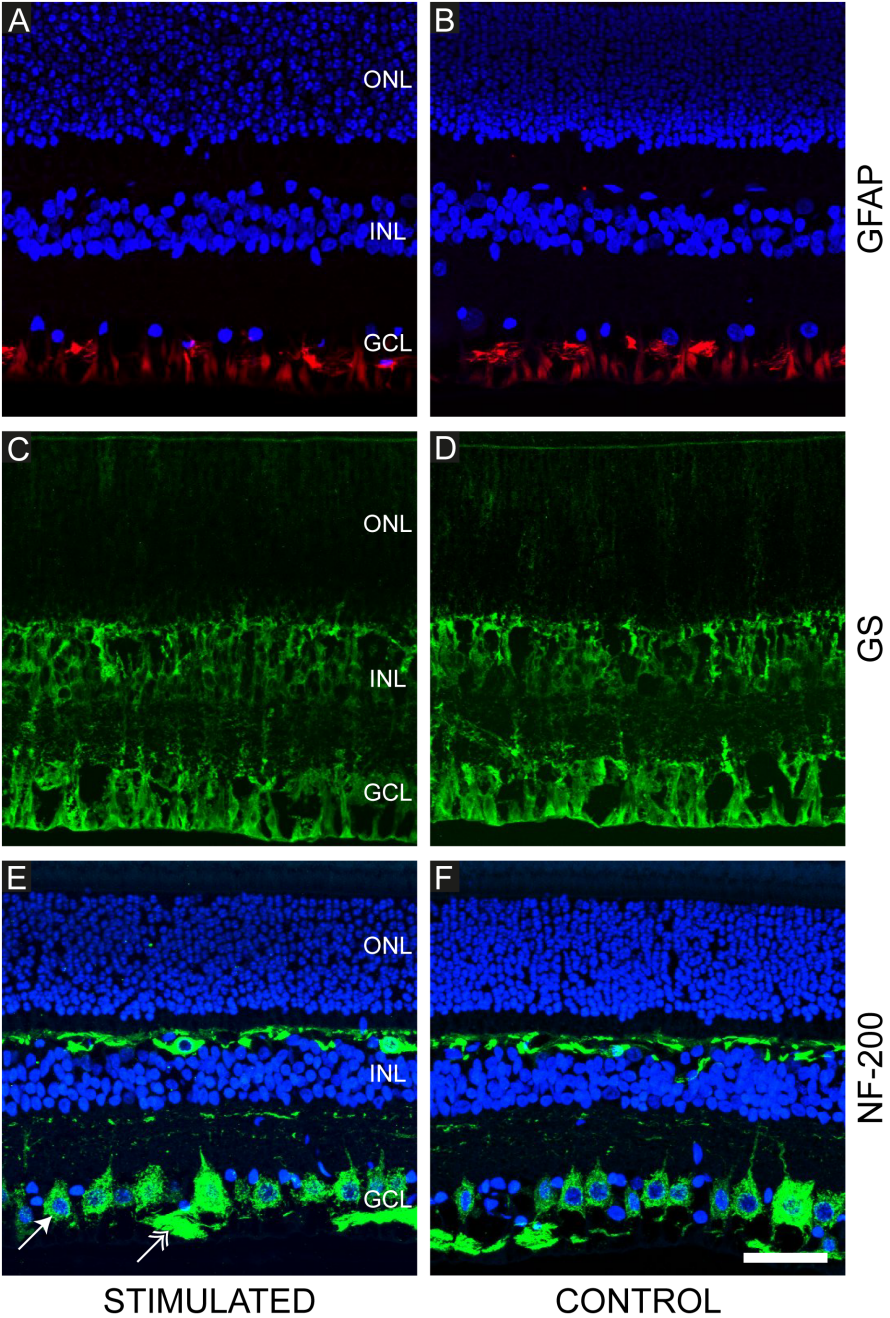
Representative immunohistochemical stains of the chronically stimulated and control feline retina. (**A, B**) anti-glial fibrillary acidic acid (GFAP; red); Müller cells and astrocytes (supporting glial cells of the retina) can be seen in the ganglion cell layer and nerve fibre layer. No evidence of gliosis was noted in any eye in the study. (**C, D**) Glutamine synthetase (GS; green); Müller cells are evident extending through the retinal layers. (**E, F**) Neurofilament (NF-200; green); large and small ganglion cells (arrow) can be seen in the ganglion cell layer with their axons forming bundles in the nerve fibre layer (double arrowhead). Horizontal cells found in the inner nuclear layer are also stained in the feline retina. Sections are counterstained with DAPI nuclear stain (blue; panels: A, B, E and F). Variations in intensity of DAPI staining are artifactual. In all cases, normal retinal architecture was observed indicating retinal cell viability. Scale bar in panel F = 50 µm and applies to all panels. The inner retina is shown at the bottom of each image; the outer retina is shown at the top of each image. Abbreviations: GCL, ganglion cell layer; INL, inner nuclear layer; ONL, outer nuclear layer. Subject ID: 507.

Immunohistochemical staining was performed on representative samples from each subject used to observe potential markers of retinal injury. Four stimulated and four control samples from each subject were examined for each of the three immunohistochemical markers. Figure 13 shows confocal microscopy images from representative stimulated and unstimulated (non-implanted control) cases (n = 168) stained with GFAP/DAPI, GS and NF-200/DAPI. Qualitative examination of these immunohistochemical samples by two consultant pathologists did not reveal any abnormalities.

#### Pathology scoring

Eighty-four dye-adjacent tissue samples (out of a possible 98) were retrieved from the 7 implanted eyes. Additionally, 85 dye-adjacent paired control samples were retrieved from the 7 non-implanted contralateral eyes. Therefore a total of 169 sites (out of a possible 196) were retrieved and scored. With the exception of 2 implanted samples which received scores of ‘1’ in the ‘Fibroblastic’ category, and 1 implanted sample which received a score of ‘1’ in the ‘Acute Inflammation’ category, all the other samples received scores of ‘0’ in all eight assessment categories (total of 1349 ‘zero’ scores). There were no clinically relevant differences in pathology scores observed between the tissue samples adjacent to maximally stimulated, partially stimulated and unstimulated electrodes. There were no differences in pathology scores between 600 µm active electrodes and 2 mm return electrodes. Furthermore, there were no differences observed in the tissue adjacent to stimulated, unstimulated or partially stimulated electrodes over and above the normal reaction to a passive implant in the suprachoroidal space [26]. Test and retest results were consistent with one another. No further analysis was conducted on these data.

## Discussion

The present study has assessed the safety and efficacy of a retinal prosthesis using a chronic stimulation model. These results demonstrated the safety and efficacy of a suprachoroidal electrode array following chronic implantation and electrical stimulation at stimulus levels of up to 77 µC.cm^−2^ pp using Pt electrodes. These subjects received continuous stimulation for periods of up to 3 months. There were no morphological abnormalities noted in fundus imagery, optical coherence tomography or histopathology. There were no observable differences in the minor scarring around the space occupied by the electrode array, fibroblastic response, or foreign body response between stimulated and unstimulated regions. There was no evidence of necrosis, acute/chronic inflammatory response or secondary retinal/remote damage in any of the samples examined. Specific stains and immunohistochemistry did not reveal any additional injuries, infection or neural damage to the retina. The thickness of the fibrous capsule which developed around the implant was minimal and consistent with previous passive studies [26]. There was no quantifiable reduction in retinal thickness adjacent to chronically stimulated electrodes, as would be expected if there was outer retinal degeneration [4], [42]. Furthermore, the clinical and histological measurements of retinal and choroidal thickness were comparable. This specific result builds confidence for the use of OCT as both a clinical and preclinical tool for retinal prostheses studies.

Evoked cortical responses to the suprachoroidal stimulation remained stable throughout the study. Longitudinal measurements of cortical evoked potentials ensured that electrical stimulation was delivered at suprathreshold levels throughout the study. This finding suggests that there were no major functional changes in the retinal ganglion cells as a result of chronic implantation and electrical stimulation. The one exception to this was subject 503, which did display an overall increase in eEVCP thresholds over the course of the study (Figure 8B). This may be due to the minor infection at the apex of the skull – near the indwelling recording electrodes. Subject 503 also showed the highest multi-unit thresholds in the terminal electrophysiology session. This was likely to be due to the superolateral placement of the suprachoroidal array. The measured eEVCP thresholds are likely related to the proximity of the stimulating electrodes to the *area centralis* of the feline retina [26] due to the large representation of the area centralis on the surface of the visual cortex [43]. Although the fact that subject 503 received the highest stimulation current should not be discounted. A follow-up histopathological and immunohistochemical examination of retinal sections from subject 503 did not reveal any noticeable differences compared with the other subjects. Further studies are required to clarify the relationship between high charge or charge density levels and reductions in neural responsiveness.

Suprachoroidal electrode arrays have already been shown to be mechanically stable during passive chronic implantation [26] and the present study confirms both mechanical and functional stability following the introduction of continuous electrical stimulation. The median electrode impedance was stable over time which is consistent with long term mechanical stability.

The present study reports results from normally-sighted subjects with intact photoreceptors. This was for two reasons. Firstly, the photoreceptor layer is a monolayer of cells which form the outermost retinal layer lying closest to the suprachoroidal space. Damage from electrical stimulus by a suprachoroidal implant would likely to be inflicted on this layer of cells [44]. Secondly, it was important to assess whether chronic electrical stimulation would have any deleterious effects on residual vision. ERG recordings revealed no reductions in retinal function following the chronic stimulation period. Furthermore, chronically stimulated eyes showed no difference in ERG a- and b-wave amplitude, latency or time course compared with the contralateral non-implanted eyes. These functional data were confirmed by histological results showing that the normal retinal architecture was preserved after suprathreshold chronic stimulation.

Clinical results using a similar suprachoroidal electrode array suggest that patient charge density thresholds range between 35–92 µC.cm^−2^ pp. It should be noted that stimulus parameters in excess of those validated in preclinical models have been needed in clinical retinal prostheses in order to obtain psychophysics and vision processing outcomes with a useful dynamic range of at least 4–6 dB above threshold [45]. The present study used monopolar electrode stimulation and did not address the safety of other modes, such as common ground, bipolar, or hexagonal [39]. Other groups running retinal prostheses clinical trials have reported mean thresholds to be 296 µC.cm^−2^ pp (n = 703; [46]) for an epiretinal device and 79 µC.cm^−2^ pp (n = 10; [47]) for suprachoroidal. The results of the present study cannot rule out stimulus-induced changes at charge densities above 80 µC.cm^−2^ pp, and rates above 200 Hz. Furthermore, in order to create controllable phosphene patterns for patients, some form of simultaneous stimulation strategy, likely involving current-steering, may be required. Stimulation parameters such as these would require additional preclinical safety studies.

Previous *in vitro* and acute *in vivo* studies have investigated the upper bounds of retinal electrical stimulation at greater levels than those examined in the present study. Direct comparisons with historical studies are complicated by the different electrodes, locations and stimulus parameters utilised in the different studies [Table 1]. A useful metric for comparison is charge density per phase which is the charge delivered per phase divided by the geometric surface area of the electrode. Ganglion cells were depressed at levels higher than 133 µC.cm^−2^ pp *in vitro* (using saline-filled hollow tube electrodes) [48] and retinal damage was observed *in vivo* at charge density levels above approximately 250 µC.cm^−2^ pp (using high real surface area, semi-porous, Pt dome electrodes) [49]. Additionally, electrical stimulation at high charge densities of 960 µC.cm^−2^ pp (using Pt wire electrodes) exacerbated the damage caused by contacting the epiretinal surface [12]. Future chronic studies are needed firstly to define the safe stimulation boundary conditions for visual prostheses, within the established electrochemical guidelines for electrode materials [20]–[22], and secondly to compare these results in a blind model.

In conclusion, using both functional and histological measures there was no evidence of injury as a result of chronic implantation and electrical stimulation of a suprachoroidal electrode array using stimulus levels known to activate the visual pathway.

## Supporting Information

Figure S1 **Example fundus image of a subject’s retina 3 months post-implantation with a suprachoroidal electrode array.**
(TIF)

Video S1 **Example X-ray fluoroscopy of a subject’s eye 3 months post-implantation with a suprachoroidal electrode array.**
(MP4)
