# Supplementary figures and images for: Chronic Electrical Stimulation with a Suprachoroidal Retinal Prosthesis: A Preclinical Safety and Efficacy Study
Source: PLoS One. 2014 May 22;9(5):e97182. doi: 10.1371/journal.pone.0097182 (PMC4031073; doi:10.1371/journal.pone.0097182)

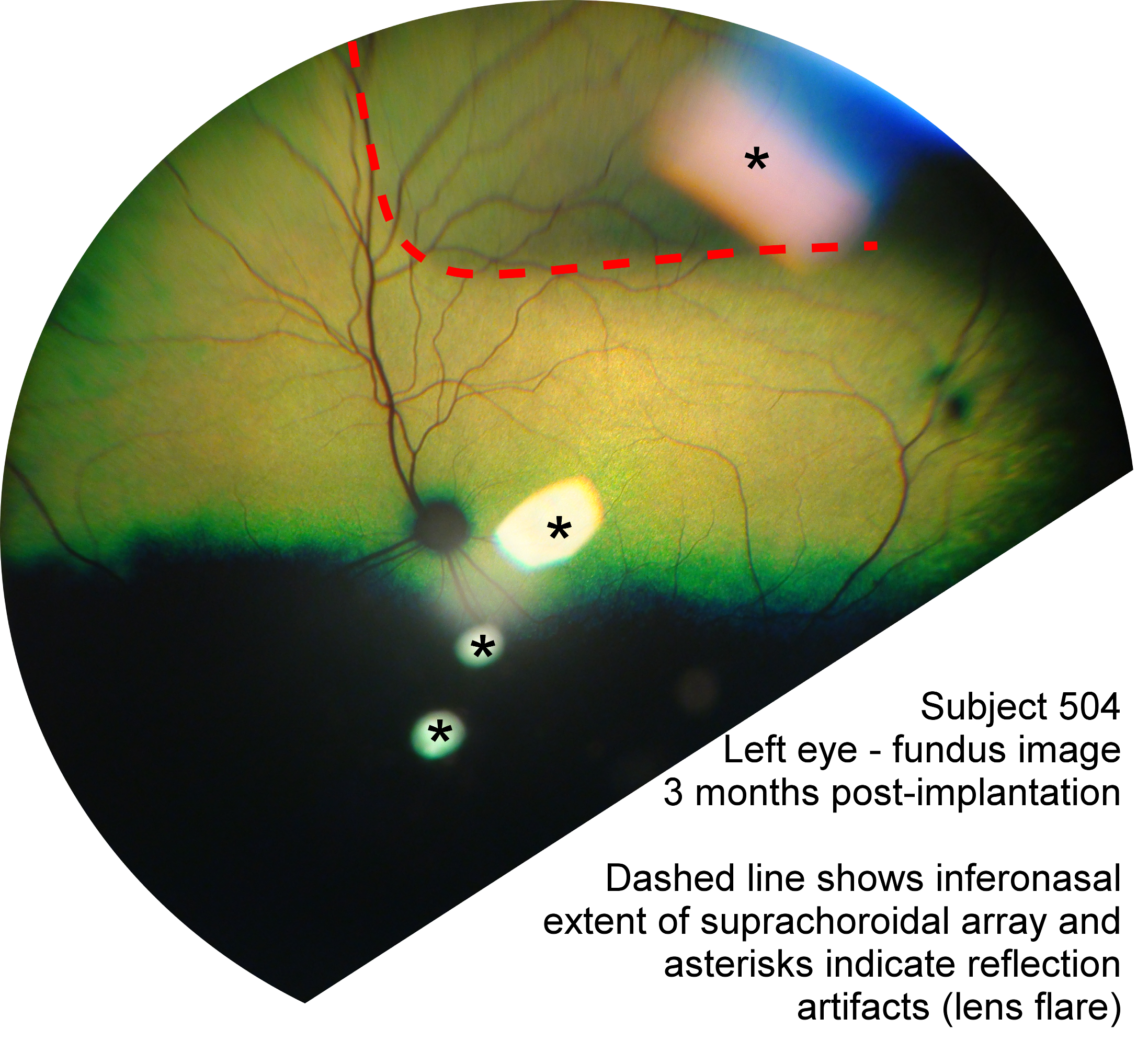

Supplement: Figure S1 — Example fundus image of a subject’s retina 3 months post-implantation with a suprachoroidal electrode array. (TIF) [file pone.0097182.s001.tif]
